# The G Protein-Coupled Receptor UT of the Neuropeptide Urotensin II Displays Structural and Functional Chemokine Features

**DOI:** 10.3389/fendo.2017.00076

**Published:** 2017-04-25

**Authors:** Hélène Castel, Laurence Desrues, Jane-Eileen Joubert, Marie-Christine Tonon, Laurent Prézeau, Marie Chabbert, Fabrice Morin, Pierrick Gandolfo

**Affiliations:** ^1^Normandie University, UNIROUEN, INSERM, DC2N, Rouen, France; ^2^Institute for Research and Innovation in Biomedicine (IRIB), Rouen, France; ^3^CNRS UMR 5203, INSERM U661, Institute of Functional Genomic (IGF), University of Montpellier 1 and 2, Montpellier, France; ^4^UMR CNRS 6214, INSERM 1083, Faculté de Médecine 3, Angers, France

**Keywords:** G protein-coupled receptor, UT, urotensin II, proline, chemokine, migration

## Abstract

The urotensinergic system was previously considered as being linked to numerous physiopathological states, including atherosclerosis, heart failure, hypertension, pre-eclampsia, diabetes, renal disease, as well as brain vascular lesions. Thus, it turns out that the actions of the urotensin II (UII)/G protein-coupled receptor UT system in animal models are currently not predictive enough in regard to their effects in human clinical trials and that UII analogs, established to target UT, were not as beneficial as expected in pathological situations. Thus, many questions remain regarding the overall signaling profiles of UT leading to complex involvement in cardiovascular and inflammatory responses as well as cancer. We address the potential UT chemotactic structural and functional definition under an evolutionary angle, by the existence of a common conserved structural feature among chemokine receptorsopioïdergic receptors and UT, i.e., a specific proline position in the transmembrane domain-2 TM2 (P2.58) likely responsible for a kink helical structure that would play a key role in chemokine functions. Even if the last decade was devoted to the elucidation of the cardiovascular control by the urotensinergic system, we also attempt here to discuss the role of UII on inflammation and migration, likely providing a peptide chemokine status for UII. Indeed, our recent work established that activation of UT by a gradient concentration of UII recruits Gαi/o and Gα13 couplings in a spatiotemporal way, controlling key signaling events leading to chemotaxis. We think that this new vision of the urotensinergic system should help considering UT as a chemotactic therapeutic target in pathological situations involving cell chemoattraction.

## Introduction

The urotensinergic system was previously considered as being linked to numerous pathophysiological states, including atherosclerosis, heart failure, hypertension, pre-eclampsia, diabetes, renal disease, as well as brain vascular lesions. Based on this expectation, validation of urotensin II (UII) receptor (UT) antagonism in cell lines expressing rat or human UT, observations in animal models, and even clinical results were not as beneficial as expected, probably because of the complex effects of the urotensinergic system depending on the vascular bed, the studied animal species, and/or the administration route. Thus, it turns out that the actions of the UII/UT system in animal models are currently not predictive enough in regard to their effects in human clinical trials, thus many questions remain regarding the overall signaling profiles of UT leading to complex involvement in cardiovascular, inflammatory responses, and cancer. We, here, propose that UII may rather play chemokine functions leading to long-term tissue remodeling and tumorigenesis, at least in part due to the pleiotropic functions of UT oriented toward chemoattractant activities.

## The UII Peptide System

### Endogenous Urotensinergic Peptide Ligands, from Gene to Sequence

At the end of the 1960s, Drs. Bern and Lederis attributed the name “urotensins” to a series of biologically active peptides isolated from the urophysis neurosecretory system of the teleost fish *Gillichthys mirabilis*. Among those, UII was characterized through its ability to stimulate smooth muscle cells (1). Then, the amino acid sequence of UII was subsequently identified in a number of other fish species, and the presence of the UII peptide was discovered in the brain of a tetrapod, the frog *Rana ridibunda* (2, 3) two decades later (Table 1). Based on these observations, the gene encoding UII has been the subject of more research and was successfully identified in various mammalian species including in monkey and human (Table 1) (4, 5). The neuropeptide UII is composed of 11 amino acids in primates (including *Homo sapiens*) to 17 amino acids in the mouse and shows remarkable conservation of the C-terminal CFWKYC hexapeptide portion formed by the covalent disulfide bridge (Table 1) during evolution, suggesting a crucial importance of the cycle in biological activity. To date, UII has been characterized in a single species of invertebrates, the *Aplysia californica* (6), in a form composed of 20 amino acids and whose cyclic hexapeptide differs from vertebrates by only two residues (F→L and Y→V) (7).

**Table 1:** **Comparison of the sequences of urotensin II (UII) and urotensin II-related peptide (URP) in different species of tetrapods**.

Family	Species		Peptide sequences	
	Scientific names	Common names	UII	URP
Tetrapods	*Pelophylax ridibundus*	Frog	AGNLSE  V(2)	
	*Hyla arborea*	Tree frog	AGNLSE  V(2)	
	*Xenopus laevis*	Xenope	GNLSE  V	A  V
	*Gallus gallus*	Chicken	GNLSE  V	A  I
	*Taeniopygia guttata*	Zebra finch	GNLSE  V	A  I
	*Felis catus*	Cat	GSPSE  V	
	*Sus scrofa*	Pig	GPPSE  V (8)	
	*Ovis aries*	Sheep	GPSSE  V	
	*Bos taurus*	Cattle	GPSSE  V	A  V
	*Rattus norvegicus*	Rat	QHGTAPE  I (5)	A  V (9)
	*Mus musculus*	Mouse	QHKQHGAAPE  I (10)	A  V (9)
	*Otolemur garmettii*	Galago	GTPSE  V	A  V
	*Callithrix jacchus*	Marmoset	ETPD  V	
	*Papio anubis*	Baboon	ETPD  V	A  V
	*Macaca mulatta*	Rhesus monkey	ETPD  V	A  V
	*Macaca fascicularis*	Macaque	ETPD  V	
	*Nomascus leucogenys*	Gibbon	ETPD  V	
	*Pongo abelii*	Orangutan	ETPD  V	A  V
	*Gorilla gorilla*	Gorilla	ETPD  V	A  V
	*Homo sapiens*	Human	ETPD  V (5)	A  V
	*Pan paniscus*	Bonobo	ETPD  V	A  V
	*Pan troglodytes*	Chimpanzee	ETPD  V (11)	A  V

*The biologically active sequence of the peptides, highlighted in red, is conserved in all tetrapods*.

All the amino acid sequences of UII identified so far are mostly deduced from cDNAs and correspond to the C-terminal part of its precursor. In human, the deduced sequence of prepro-UII, cloned from colon tumor or placental library, evolved from alternative splicing of the human UTS2 gene, yielding a 124 (isoform b, NP_006777) and 139 (isoform a, NP_068835.1) amino acid variants. The two encoded isoforms are identical for the last 97 amino acids but differ at their N-terminal end exhibiting the signal peptide. The mature peptide UII results from the proteolysis of preproprotein UII at the tribasic site KKR by a specific urotensin converting enzyme (UCE), which is not still identified (4, 5). Study on the conversion of a 25 amino acid C-terminal fragment of preproprotein to mature peptide revealed that the endoprotease Furin and the serine protease trypsin, may act, respectively, as intracellular and extracellular UCE (12). This enzymatic cleavage appears necessary to confer biological activity (13).

In comparison with primate prepro-UII, precursors of rat and mouse UII markedly diverge by the amino acid composition of the N-flanking domain of the cyclic hexapeptide and by the absence of a typical cleavage site (KKR) for pro-hormone converting enzymes in the upstream region of UII sequence (10). These observations led Sugo and collaborators to characterize UII immunoreactivity detected in the brain of the two rodent species and to isolate, in 2003, a peptide similar to UII, the urotensin II-related peptide (URP) (9). Later on, the cloning of the prepro-URP cDNAs, in human, mouse, and rat revealed 54% homology between human and rat vs 47% homology between human and mouse (14). However, the URP sequence is identical in all mammals and corresponds to human Ala^1^–UII_4–11_. Finally, although URP was initially thought to exist only in tetrapods, its gene has been identified in the genome of several teleost fishes (15, 16). Together, the sequences of UII, URP, and somastostatin display high homology in particular at the level of the cyclic hexapeptide sequence and it was established that URP is a peptide paralog of UII (17).

### General Distribution of UII and Urotensin II-Related Peptide

Urotensin II and URP are widely distributed in the cardiovascular, renal, and endocrine systems. In humans, high expression levels of UII are found in the myocardium (18), the atria, and the ventricles (19–21). UII has also been detected in the heart of rats (4, 9, 20) and mice (11, 22). At the vascular level, the presence of mRNA for prepro-UII has been demonstrated in the arterial network, primarily in the thoracic aorta, pulmonary arteries, and arterioles. In contrast, it is almost absent in the venous network, with the exception of the saphenous and umbilical veins (19–21).

Several studies show that kidney is a major site of production of circulating UII in humans (9, 20, 21, 23, 24). The peptide is particularly abundant in glomerular epithelial cells, convoluted tubules, and collecting ducts (20, 25). Surprisingly, the level of expression of UII in the kidney of monkey and mice is weak (11), stressing some important differences between species. UII is also expressed in endocrine glands, such as pancreas or adrenal gland, in humans and rats (5, 23, 26). Nevertheless, the mRNA for UII is undetectable in these tissues in monkey and mice (11, 22), again raising the question of the occurrence of a conserved cardiovascular and/or endocrine role of UII among the different species.

Even though the identification of URP has been done more than 10 years ago, data concerning this peptide are considerably much more incomplete. Nevertheless, it is worth noting that the expression of prepro-URP is predominant in the gonads and placenta of humans and in the testis of rats (9). URP and its mRNA are also expressed in kidney (8, 9) and in the ventricles and myocardium of the rat heart (27, 28). The expression of the two peptides extends to the spleen, thymus, liver, stomach, and intestines (5, 9, 11, 20, 22, 23, 29, 30).

Within the central nervous system (CNS), UII immunoreactivity is mainly associated with motoneurons of the hypoglossal nucleus of the brainstem and the ventral horn of the spinal cord. This neuronal subpopulation also strongly expresses UII in the nuclei of the abducens, facial, trigeminal, and hypoglossal cranial nerves in rats (10, 31) and those of the caudal part of the spinal cord in mice (10, 32), rats (10, 31), and humans (4, 5). Surprisingly, UII is apparently absent from the brainstem of monkey (4, 11). URP mRNAs are localized in the spinal cord of humans and rats, at expression levels considerably lower than those of UII (9). In mice, URP mRNA is found in the brainstem and in motoneurons of the anterior horn of the spinal cord (22). Finally, URP is present in neuronal cell bodies of the preoptic region and in fibers of the median eminence and the organum vasculosum of the lamina terminalis, which is involved in thermoregulation (33).

Thus, UII and URP are not ubiquitously expressed within the peripheral and central nervous systems and likely show key expression levels in heart, arterial networks, and kidney with discrepancies between species, suggesting a non-conserved role in the vasomotor tone regulation.

## UII Receptor UT Reconsidered in Light of Conserved Structural Properties

The UT receptor was initially discovered and cloned in 1995 from rat sensory tissue extracts (34) and a rat genomic library (35). At this stage, this G protein-coupled receptor (GPCR) was named sensory epithelium neuropeptide-like by Tal et al. (34) and GPR14 (according to the current nomenclature) by Marchese et al. (35). Whereas Ames et al. identified the UII peptide as the endogenous ligand of the human receptor homologous to GPR14 by reverse pharmacology (4), other research teams in the same year corroborate the existence of the UII/GPR14 system in various species (8, 36, 37). It is on the basis of these studies that the receptor was renamed UII receptor or UT, by the International Union of Basic and Clinical Pharmacology (IUPHAR).

### Distribution of UT Varies Depending on Species and Systems

The presence of substantial amounts of UII in the cardiovascular system has led several groups to investigate the expression of UT mRNA in different component tissues in rat (37–39) and mouse (36). In human and monkey, high levels of mRNA-encoding UT have been detected in the myocardium (18), the atria (4, 11, 21, 23), and the ventricles (4, 20, 23). At the vascular level, the presence of UT has been detected in the thoracic aorta (4, 21, 40) as well in the pulmonary and coronary arteries (41). In addition, UT, like UII, is strongly expressed in kidney from rat (27, 38, 42–46) and human (21, 23, 24, 41, 47), although it is only moderately expressed in monkey (44). UT is also present in the endocrine system, notably in the pituitary, pancreas, and adrenal gland in human (4, 23), monkey, and mice (11). Other peripheral tissues show significant levels of UT expression, which varies according to the species studied. The CNS shows widespread expression of UT mRNA, which is particularly abundant in the brainstem and spinal cord (23, 24, 36, 38, 48).

Other regions of the CNS, e.g., the cortex, hypothalamus, and thalamus, display relatively weak expression levels that vary between species. UT is also associated with cerebral blood vessels and is expressed mainly in the endothelial cells of microvessels (49). Finally, the expression of the receptor has been detected both in neurons (48) and in a subpopulation of astrocytes in the brainstem and hypothalamus (50) and in cultured cortical astrocytes (51).

Together, this UT distribution highly resembles the UII/URP distribution in cardiovascular endocrine and also nervous tissues, naturally leading several groups to investigate the effects of UT on the cardiovascular system, even if the data remain multiple and complex. In human, circulating levels of UII and/or URP (“UII-like”) are higher in patients with heart failure (52, 53), systemic (54) or portal hypertension (55, 56), or atherosclerosis (57), than in plasma of healthy volunteers. In fact, the UT-related mechanisms appear associated with tissue remodeling processes during the course of the disease (58), including cardiac hypertrophy and fibrosis (59). Thus, we here question whether UT may play an alternative chemokine-like function in primates than vasomotor regulatory activities as previously proposed in rats.

### The UT Positioning Depends on the Different GPCR Classifications

Although GPCRs share a common structure, certain characteristics make it possible to distinguish and to classify them in different families. However, based on the homology of sequence, structure, ligand binding mode, or phylogenetic relationships, the large number of GPCRs makes it difficult to develop a global classification system. The human UT receptor was shown to belong to the class A (Rhodopsin) GPCR family (60) according to the widely used structural classification in the past, based on the identification, by analysis of protein sequences of the TMs of the GPCRs listed in vertebrates and invertebrates, of fingerprints preserved within certain GPCR groups (61). GPCR members of class A (the largest family of GPCRs with 80% of GPCRs listed) share homologies of sequence, structure, and ligand-binding mode. The homologies of sequence between the receptors of class A can be very low since they rely on the conservation of a few residues mainly located in the TMs, which would play a primordial role in their structure and functionality. Within this classification, UT displays sequence homology not only with certain somatostatin receptors (SST), in particular with SST4 (27%), but also opioïdergic receptors (MOR: 26%, DOR: 26%, and KOR: 25%) (35), which are now crystallized (62, 63) and would constitute the best prototypes for UT modeling.

More recently, Fredriksson et al. (64) proposed from the GPCR sequences a yet commonly used systematic classification system named GRAFS formed by the five distinct families of *Glutamate* (G), *Rhodopsin-like* (R), *Adhesion* (A), *Frizzled/Taste* (F), and *Secretin* (S). The Rhodopsin-like family showed a clear evolutionary success since containing around 90% of the GPCRs and is divided into four (α, β, γ, and δ) subclasses in Fredriksson’s classification. The crystallographic structures of Rhodopsin-like family indicate a common firm core corresponding to high conserved sequence motifs, i.e., E/DRY in TM3, NPXXY on TM7, WXP on TM6, D2.50 in TM2 (*X*.50, according to the Ballesteros classification: *X*, numbering of TM; 50, the most conserved residue in the concerned TM) (65), and a water network that can be seen in the binding pocket mediating ligand interactions with the receptor (66). It can be noticed that the γ group includes 59 GPCRs, divided into three different clusters, i.e., SOG (15 GPCRs like SST, OR or GPR54 receptor also named KISS1R), melanin-concentrating hormone receptors (MCHR) (2 GPCRs), and CHEM (42 GPCRs) including chemokine receptors, such as the CXCR4, angiotensin (ANG), and bradykinin (BK) receptors, as well as a large number of orphan receptors. However, the neighbor-joining and maximum parsimony method used in sequence analysis failed to affect 23 receptors into one family/group/cluster, and this is the case for UT (named GPR14 in this study). These difficulties were due to an unusual part of the GPCR gene sequence in question [usually coding for intra- (i) or extracellular (e) loops] that would result from a chimeric origin of the receptor and/or progressive pressure not shared by neighboring receptors (64). However, UT shares the sequence pattern characteristic of Rhodopsin-like GPCRs. When comparing different sub-families of GPCRs from the conserved ligand binding pocket or from conserved endogenous agonist ligands (67), UT can be found near GPR109A or purinergic P2Y receptors listed in orphan receptors from the SOG or PUR cluster group by Fredriksson et al. (64, 67). In light of these results, we suggest that UT possesses specific structures and functions related to the chemokine receptors of the SOG and PUR families.

**Figure 1:**
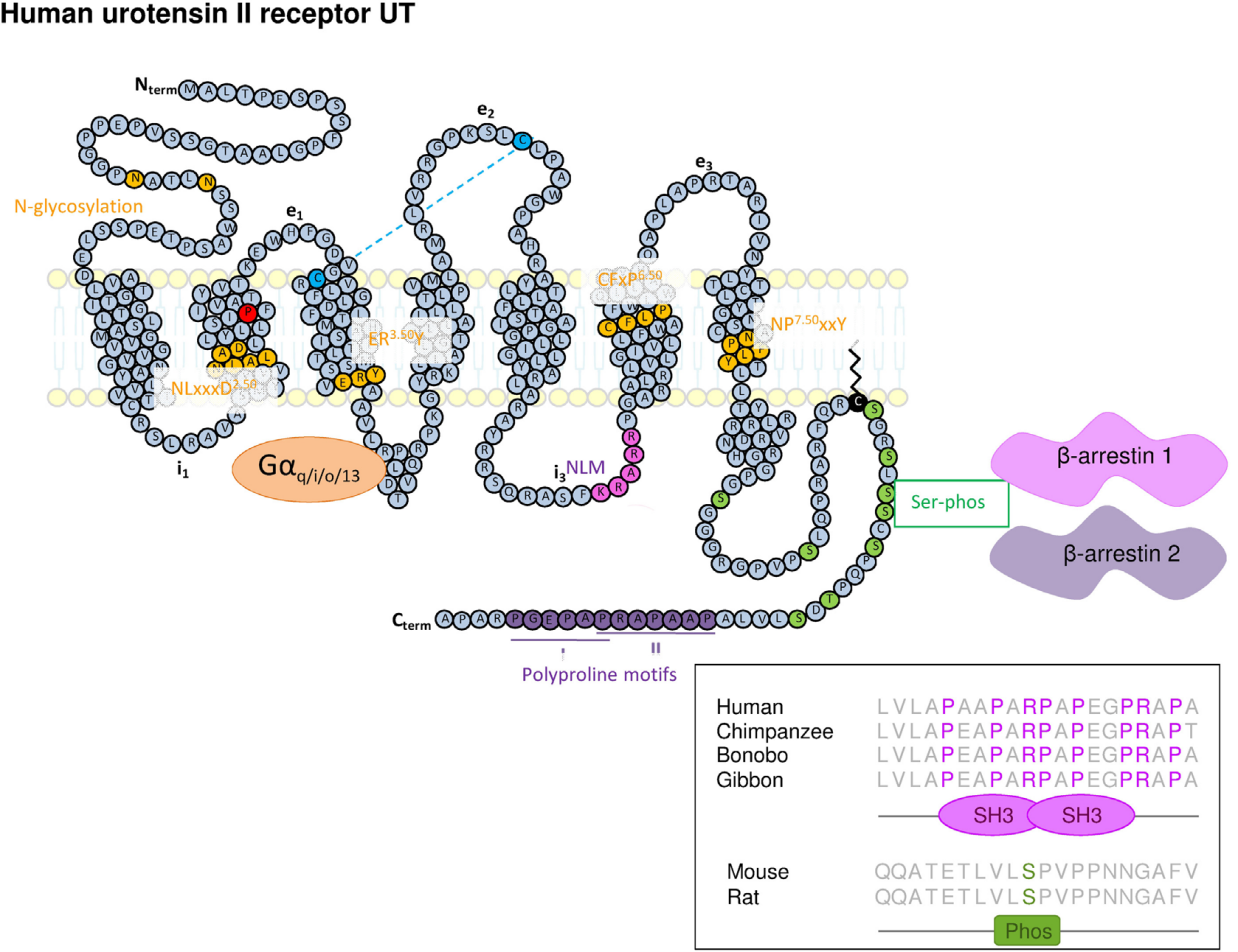
**Schematic representation of the structure of human UT**. The amino acids represented in yellow represent highly conserved residues within class A/Rhodopsin G protein-coupled receptor of which the UT is belonging. It concerns two N-glycosylation sites in the N-terminal part (Nterm), a NLxxxD motif in TM2, a ERY motif at the cytoplasmic end of the TM3, the CFxP motif in TM6, and NPxxY within the TM7. The key proline in position 2.58 appears in red within the TM2. The two cysteine residues involved in the disulfide bridge between the extracellular end of the TM3 and the e2 loop appear in blue. A nuclear localization motif (NLM) sequence (in pink) was also identified in i3 loop. In addition to these consensus motifs, the C-terminal tail of UT exhibits Serine phosphorylation sites (in green) potentially involved in β-arrestin 1 and 2 anchoring, cysteine, palmitoylation site and plasma membrane anchor (black) sites, as well as two polyproline type I and II motifs (in violet) extracted from analysis by Scansite (http://scansite3.mit.edu#home). Inset, the alignment of the UT C-terminal (C-term) sequence shows that the prolyproline motif allowing interaction with SH3 protein domain, is specifically conserved in hominoids.

Thus, as members of the Rhodopsin SOG and PUR family, UT has a relatively short N-terminal domain with two N-glycosylation sites (N29 and N33), a NLxxxD2.50 motif within its TM2, a disulfide bridge between cysteine residues in the extracellular end of TM3 and the e2 loop, a ER3.50Y motif at the cytoplasmic end of the TM3, a CFxP6.50 motif within the TM6, the highly conserved NP7.50xxY motif at the TM7 level, and a palmitoylation site at the C-terminal tail (C334) (68). Other specific motifs are observed, namely (i) a KRARR nuclear localization motif at the i3 loop (69), (ii) potential sites of phosphorylation by protein kinases A and C, kinase I, and glycogen synthase kinase 3 at i2 and i3 loops (35, 68, 70), (iii) serine potential phosphorylation sites at the C-terminal end involved in β-arrestin interaction and internalization of the receptor (71, 72), and (iv) polyproline type I and II motifs within the C-terminal tail potentially allowing the interaction with proteins harboring src homology 3 type domains (Figure 1).

### UT Shares a Structural Feature with Chemotactic GPCRs: An Evolutionary Lighting

These human GPCR classifications were proposed from constructions of phylogenetic trees, which require the use of several methods to assess the robustness of the obtained results. However, other strategies should be used when a position dependency hypothesis is questioned, the size of the dataset becomes large, and/or the relationships between proteins of the same family but of different genomes must be compared. Thus, to address some ambiguities concerning GPCRs classification, as highlighted for UT, analyses of gene sequences by the metric multidimensional scaling (MDS) were conducted. MDS is also called “principal coordinates analysis” and corresponds to an exploratory multivariate procedure designed to identify patterns, within proteins for example, in a distance matrix (73, 74). With MDS, protein sequences can be considered all at once, and individually represented in a low-dimensional space whose respective distances best approximate the original distances. In addition, MDS allows the projection of supplementary information allowing a straightforward comparison of the active and supplementary data. Therefore, MDS was used to explore the sequence space of GPCR families and to interpret patterns in relation with evolution, with projection of GPCR sequences from distant species onto the active space of human GPCRs (75, 76), based on the assumption that GPCR evolution could follow a radial rather than bifurcated path (represented by the classical phylogenetic tree system). The phylogenetic links between GPCRs of the same species were represented in three dimensions, and the results were shown superimposed between several species (75). By means of this evolutionary-based classification, the work of the Chabbert’s group succeeded in identifying GPCRs of the Rhodopsin class in the same clusters as those found by Fredriksson et al. (76), but some differences at the margin were also identified and likely stressed the way how some GPCRs may be activated and function. The differences are as follows: galanin receptors and Kiss1R belonging to the SOG cluster in the Fredriksson et al. classification, are likely rather connected to the PEP cluster according to Chabbert et al., and then SOG becomes SO cluster. In addition, the MCHR and UT appeared in this new classification, grouped in this SO cluster (76).

This is probably the evolutionary point of view that gives the best indications about UT membership and structural characteristics. Indeed, MDS analysis of GPCRs of the Rhodopsin family allowed the receptors to be sorted into four groups (G0–G3) comprising different clusters (76). The group G0 represents the central group and includes the clusters PEP, OPN, and MRN, the group G1 includes the cluster SO (SST, OR, and UT), CHEM, and PUR (Table 2), the group G2 contained AMIN and AD clusters and finally G3 involves LGR, MEC, PTG, and MRG clusters (Table 2). It is interesting to note that in *C. intestinalis*, the CHEM cluster only slightly differs from the SO cluster, thus suggesting that this SO/CHEM group gave rise, in vertebrates, to SO, CHEM, and PUR clusters, suggesting a common origin. Moreover, the cluster SO and PEP are close in the most distant ancestral species from human and their distance increases during evolution (75). These observations argue in favor of a common origin between PEP and SO, CHEM, and PUR clusters (76) and allow the repositioning of UT from a “peptide family (PEP)” group to a chemokine receptor family.

**Table 2:** **Assignment of the 13 non-olfactory human G protein-coupled receptor clusters from the rhodopsin class into four groups, G0, G1, G2, and G3, in addition to an UC**.

Group	Family	Pattern	*Homo sapiens*
G0	PEP	P2.58	MTLR, GHSR
	*Peptide*	P2.59	NMUR1, NMUR2, NTR1, NTR2, GPR39, EDNRA, EDNRB, ETBR2, GPR37, PKR1, PKR2, NPY1R, NPY2R, NPY4R, NPY5R, BRS3, GRPR, NMBR, CCKAR, GASR, QRFPR, OX1R, OX2R, NPFF1, NPFF2, PRLHR, GNRR2, GNRHR, GPR83, GALR1, GALR2, GALR3, KISSR, GP151, GP173, GPR19, GPR27, GPR84, GPR85
		P2.60	V1AR, V1BR, V2R, OXYR, TRFR
		NoP	NK1R, NK2R, NK3R, GP150
	
	OPN	P2.59	OPN4, OPSX
	*Opsin*	P2.60	OPSB
		NoP	OPN3, OPN5, RGR, OPSR, OPSD
	
	MTN *Melatonin*	P2.59	MTR1A, MTR1B, MTR1L

**G1**	**SO** *Somatostatinergic opioïdergic*	**P2.58**	OPRM, OPRD, OPRK, OPRX, SSR1, SSR2, SSR3, SSR4, SSR5, NPBW1, NPBW2, **UT**, MCHR1, MCHR2
	
	**CHEM** *Chemokine*	**P2.58**	CCR5, CCR2, CCR3, CCR1, CCR4, CCR8, CX3C1, CCRL2, CCBP2, XCR1, CCR9, CCR7, CCR6, CCRL1, CXCR4, CXCR2, CXCR1, CXCR5, CCR10, CXCR3, CXCR6, CXCR7, RL3R1, RL3R2, ADMR, AGTR1, AGTR2, BKRB1, BKRB2, APJ, GPR25, GPR15, C5ARL, C5AR, C3AR, GPR44, FPRL1, FPRL2, FPR1, LT4R1, LT4R2, CML1, GPR32, GPR33, GPR1
		NoP	GP152
	
	**PUR** *Purinergic*	**P2.58**	P2RY1, P2RY2, P2RY4, P2RY5, P2RY6, P2RY8, P2RY9, P2Y10, P2Y12, P2Y13, P2Y14, PTAFR, SUCR1, OXER1, OXGR1, G109A, PSYR, SPR1, CLTR1, CLTR2, PAR1, PAR2, PAR3, EBI2, FFAR1, FFAR2, FFAR3, GPR4, GPR17, GPR18, GPR20, GPR31, GPR34, GPR35, GPR55, GPR81, GPR87, GPR92, GP132, GP141, GP174, GP171, Q5KU21, GPR82
		P2.58P2.59	P2Y11, PAR4

G2	AMIN *Aminergic*	P2.59	5HT1B, 5HT1D, 5HT1E, 5HT1F, 5HT1A, 5HT7R, 5HT4R, 5HT2A, 5HT2C, 5HT2B, 5HT5A, HRH1, HRH2, HRH3, HRH4, DRD1, DRD2, DRD3, DRD4, DRD5, ADA1A, ADA1B, ADA1D, ADA2A, ADA2B, ADA2C, ADRB1, ADRB2, TAAR1, TAAR2, TAAR3, TAAR5, TAAR6, TAAR9
		P2.59P2.60	5HT6R, ADRB3
		NoP	TAAR8, ACM1, ACM2, ACM3, ACM4, ACM5
	
	AD *Adrenergic*	P2.59	AA2AR, AA2BR, AA1R, AA3R

G3	LGR *Glycoproteins*	NoP	LGR4, LGR5, LGR6, RXFP1, RXFP2, TSHR, LSHR, FSHR
	
	MEC *Melanocortin* *Cannabinoid*	NoP	ACTHR, MSHR, MC3R, MC4R, MC5R, CNR1, CNR2, EDG1, EDG2, EDG3, EDG4, EDG5, EDG6, EDG7, EDG8, GPR3, GPR6, GPR12
	
	PTGR	P2.59	PE2R2, PE2R3, PE2R4, PD2R, PI2R
	*Prostaglandin*	NoP	TA2R, PF2R, PE2R1
	
	MRG *Mas-related*	NoP	MAS, MAS1L, MRGRF, MRGX1, MRGX2, MRGX3, MRGX4, MRGRD, MRGRE

UC	UC	P2.58	GPBAR, GP120, Q5KU14, GP146
		P2.59	GPR22, GPR26, GPR45, GPR61, GPR62, GPR63, GPR75, GPR78, GPR88, GP101, GP135, GP161, GP176
		P2.60	GPR21, GPR52

*Abbreviations of the SO, CHEM, and PUR clusters of the G1 group displaying a P2.58 are in bold. The SO family containing UT is shown in red. The receptors with their most common abbreviations belonging to each of the clusters in the G0–4 and unclassified group (UC) groups are listed [from Pelé et al. (76)]*.

**Figure 2:**
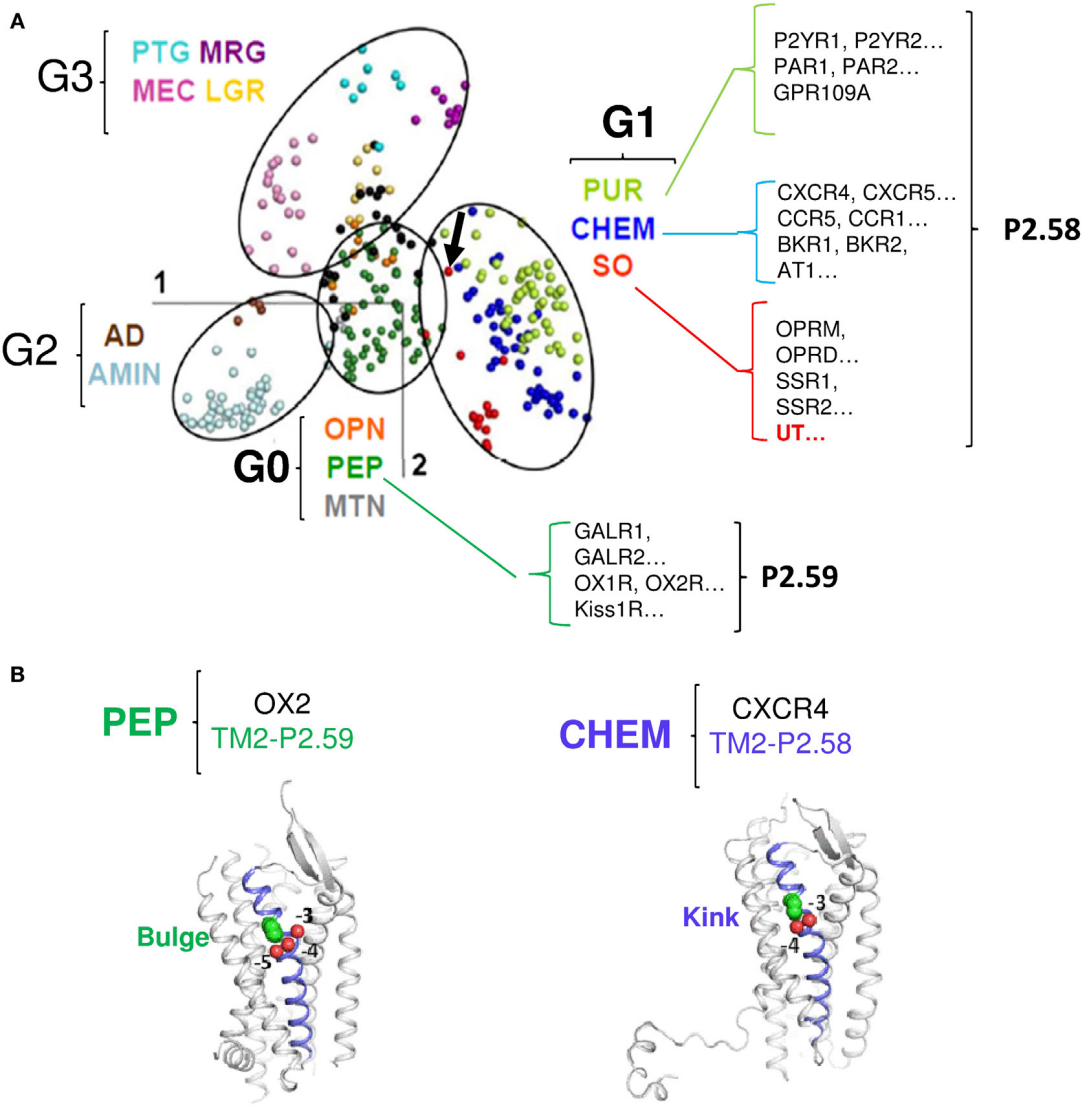
**Classification of the different G protein-coupled receptor (GPCR) sub-families according to the multidimensional scaling (MDS) analysis and focus on the proline position in TM2 of receptors from the G0 and G1 groups**. **(A)** In the MDS representation of Rhodopsin-like GPCRs, receptors are visualized as points, with the distances between points as close as possible to the distance in the identity matrix [from Ref. (76)]. The points cluster into four groups, highlighted by ellipses. The color code indicates receptor sub-families and is given in the Figure along with the group the sub-family belongs to. Examples of receptors with the position of the TM2 proline are shown for the G0 and G1 groups. The arrow indicates the position of UT [modified from Ref. (76)]. **(B)** Cartoon view of the PEP receptor OX2 (P2.59, PDB access number: 4S0V, *left panel*) and of the CHEM receptor CXCR4 (P2.58, PDB access number: 3ODU, *right panel*). TM2 is slate. The TM2 proline (green) and the preceding oxygen (red) are shown as spheres. In CXCR4, P2.58 is close to the carbonyl groups at positions −3 and −4 (proline kink). In OX2, P2.59 is close to the carbonyl groups at positions −4 and −5 (proline bulge). Thus, according to the position 2.58 or 2.59 of the TM2 proline, the structure of TM2 presents a kink or a bulge.

Sequence comparison of the different groups (G0–G3) shows that the main characteristic of the G1 group receptors, including UT is a proline within the TM2 in position 2.58 (P2.58), often preceded by an aliphatic residue whereas G0 group mainly comprises receptors harboring a proline in position 2.59. Together, only the G1 group, which includes the SO containing UT, CHEM, and PUR clusters, is therefore characterized by a proline P2.58 (75, 76) (Figure 2A). Given the phylogenetic links between the PEP, SO, CHEM, and PUR clusters, it is proposed that the position of the proline in 2.58 for the SO, CHEM, and PUR clusters results from a codon deletion in the TM2 of receptors of the PEP family. This proline in TM2 either on P2.58 or P2.59 induces a typical elbow observable by modeling (77, 78) and confirmed by crystallographic studies (79–81), yielding bulge and kink structures, in P2.59 and P2.58 receptors, respectively (Figures 2A,B). In fact, by plotting the curvature and flexibility of the TM2, the position of the proline could affect the degree of opening of the GPCR-binding pocket and their activation mechanisms (82). Thus, the change in conformation of the TM2, following the deletion of a residue within the TM2 helix, would contribute to the emergence of activation mechanisms specific to SO, CHEM, and PUR cluster receptors.

As many CHEM and PUR receptors are widely recognized as mediating chemotaxis and chemoattractant behaviors, we propose that the P2.58 and kink feature the TM2 of UT, has allowed the capacity of UII gradient sensitivity and chemotactic behavior, leading to cell migration and invasion.

## UII/UT System, from Cardiovascular Functions to Chemokine Properties

### Physiological and Pathophysiological Effects of the Urotensinergic System on the Cardiovascular Functions

The distribution of UT and its endogenous ligands has naturally led several groups to investigate the effects of UT on the cardiovascular system. When applied to de-endothelialized aortic rings from rats (4, 36, 83, 84), rabbits (85), macaques (4), or humans (20, 41, 42, 86–88), UII induces dose-dependent constriction. This effect is observed at doses so low that this neuropeptide was considered the most potent naturally occurring vasoactive compound (4, 20). For example, in a murine model, UII is 660 and 16 times as powerful as serotonin and endothelin, respectively (4). This vasoconstrictive activity is primarily relayed by the mobilization of cytosolic calcium (4, 36). Calcium recruited by UT is derived partly from an intracellular pool *via* the activation of channel receptors sensitive to inositol triphosphate (IP_3_) and partly from the extracellular pool *via* L-type calcium channels (89–92). Calcium activates calmodulin, whose blockade inhibits the effects of UII on the contraction of rat aortic rings (89). Calmodulin in turn activates myosine light-chain kinase, responsible for the phosphorylation of MLC-2 and the contraction of actomyosin (93, 94). In the sidelines of this principal intracellular signaling pathway, other pathways involved in the contractile activity of UII, such as the PKC/ERK and the RhoA/ROCK pathways, have also been identified (92–95).

However, when injected as an intravenous bolus in anesthetized or conscious rats, UII and URP provoke a slow and prolonged decrease in arterial pressure due to vasodilatation (9, 96–98). In contrast, chronic administration of UII to these animals has no effect (99). In primates, intravenous administration of UII exerts a strong vasodilatation, responsible for cardiovascular collapse and cardiac arrest at high doses (4, 100). However, results in humans are more controversial, since the intravenous injection of UII leads to local vasoconstriction (101) or has no apparent effect (102–104). Studies investigating skin microcirculation even showed that UII infusion through iontophoresis induces a dose-dependent vasodilatation in healthy volunteers but a dose-dependent vasoconstriction in patients with chronic heart failure, systemic hypertension, cirrhosis, or diabetes without cardiovascular pathology (54, 105–107). Finally, endothelium alterations observed in these pathologies could alter vasodilator properties of UII and explain, at least in part, the differences between patients and healthy volunteers.

Overexpression of UII, URP, and UT in the heart of rats and humans with heart failure has also been demonstrated (13, 28) with a correlation between UII plasma level and the cardiac dysfunction (108). A strong “UII-like” immunoreactivity was seen in coronary artery endothelial cells from patients with atherosclerosis (20, 109), associated with a significant effect of UII on the proliferation of vascular smooth muscle cells (95, 110) or the formation of foam cells (111, 112). Moreover, in rat models, treatment by a UT antagonist reduces mortality and improves cardiac function after myocardial infarction (113), decreases coronary angioplasty restenosis (114), pulmonary arterial hypertension (115) and aortic inflammation, and atherosclerosis (116).

Taken together, these data suggest that this peptide could participate rather in tissue remodeling processes during the course of the vascular disease (58) than in tonic vasculo-motor functions. This hypothesis is reinforced by the absence of modification of the vascular tone, and the appearance of a reduced metabolic syndrome and atherosclerotic lesions in UII knockout in comparison with wild-type mice (117).

### Effects of UII on Cell Proliferation, Survival, and Hypertrophy

More related to tissue remodeling, the urotensinergic system exerts promitogenic effects on a number of native and recombinant cell types and hypertrophic functions only on cardiomyocytes (Table 3). The activation of ERK is a central element of these effects, either in cell lines transfected with cDNA encoding human UT (118) or in native cells expressing the receptor, i.e., pig renal epithelial cells (119) or rat smooth muscle cells (120). Several signaling pathways leading to the activation of ERK and cell proliferation, survival, or hypertrophy have been described in the literature. One of these pathways involves the transactivation of the epidermal growth factor receptor (EGFR) (121–123). This is often dependent on the production of reactive oxygen species (ROS) by an NADPH oxidase activated by UT (124). The ROS relieve the inhibition exerted by src homology 2-containing tyrosine phosphatase (SHP-2) on EGFR, allowing the transduction of the mitogenic signal (123, 125). This phenomenon of transactivation can also be underpinned by the activation of a disintegrin and metalloproteinase (ADAM) which cleaves the precursor of EGF, the heparin-binding EGF-like growth factor, and releases the active ligand EGFR accordingly (122, 126) (Table 3). The promising effects of UT are also relayed by other second messengers than previously described (PLC and PI3K), *via* receptor coupling to a pertussis toxin-sensitive G_i/o_ proteins in native (45, 118, 127, 128), tumoral human rhabdomyosarcoma (129), or recombinant cell lines (130). These last observations suggest that the ability of UT to coupled G_i/o_ in addition to G_q_, may have provided acquisition of specific skills important for other activities than cardiovascular tone regulation.

**Table 3:** **Transduction pathways associated with UT receptor activation and involved mitogenic and chemokine functions other than cardiovascular tone regulation**.

Effect	Cell type	Species	Transduction pathways	Reference
Proliferation	Arterial SMC	Rabbit	PKC, src, MAPK	(110)
		Rat	RhoA, ROCK	(95)
	CHO-UT	Hamster	G_i/o_, PI3K, PLC, calmodulin, MEK, extracellular Ca^2+^	(118)
	Renal epithelial cells	Pig	Ca^2+^ (voltage-dependent channels), PKC, MAPK, ERK, c-myc	(119)
	Airway SMC	Rat	PKC, MAPK, Ca^2+^, calcineurin	(131)
	Cardiac fibroblasts		EGFR transactivation, ERK, ROS	(121)
	Renal tubular cell line		ROS, inhibition of SHP-2, EGFP transactivation *via* HB-EGF	(122)
	SMC		Ca^2+^, CaMK, ERK, PKD	(132)
	Endothelial precursors		ERK, p38MAPK	(133)
	Airway SMC		ERK, TGFβ	(120)
	Airway SMC	Human	NOX, ROS, ERK, p38MAPK, c-Jun, Akt, expression of PAI-1	(124)
			NOX4, ROS, FoxO3, JNK, MMP-2	(134)
	Astrocytes	Rat	PLC, intra- and extracellular Ca^2+^ (T-type channel), IP_3_, G_i/o_	(128)
	Fibroblastes		MAPK, VEGF expression, collagen production	(135)
	Aortic SMC		ROS, SHP-2 inhibition, EGFR transactivation	(123)
	HUVEC	Human	p38MAPK, ERK	(136)
	Cardiac precursors	Mouse	JNK, LRP6	(137)

Survival	Vascular SMC	Rat	N. D.	(138)
	Cardiomyocytes		PI3K, ERK	(139)

Hypertrophy	Cardiomyocytes-UT	Rat	G_q_, Ras	(59)
			EGFR transactivation *via* HB-EGF, ERK, p38MAPK	(126)
	Cardiomyocytes		ROS, SHP-2 inhibition, EGFR transactivation	(125)
			PI3K, Akt, GSK-3β	(140)
			ROS, NADPH oxydase, Akt, GSK-3β, PTEN	(141)

Angiogenesis	HUVEC	Human	PLC, Ca^2+^, PKC, PI3K, ERK1/2, FAK	(142)
			VEGF, endothelin-1 and adrenomedullin expression	(143)
			HIF-1, ROS, NOX-2	(144)
	Neuromicrovascular endothelial cells	Rat	N.D.	(49)
	Chick embryo chorioallantoic membrane	Chicken	N.D.	(49)

Migration, motility, adhesion	HEK293	Human	N.D.	(130)
	Monocytes	Human	RhoA, ROCK	(145)
	Endothelial progenitors	Rat	RhoA/ROCK, MLC	(146)
	Prostatic adenocarcinoma (LNCaP)	Human	RhoA, FAK	(147)
	Vascular SMC		MEK	(148)
	Vascular fibroblasts	Rat	PKC, ROCK, calcineurin, MAPK	(149)
				(150)
	Endothelial progenitors		N.D.	(151)
	Colorectal carcinoma	Human	N.D.	(152)
	Bladder cancer		N.D.	(153)
	Glioblastoma cell line		G_13_/Rho/ROCK, G_i/o_/PI3K	(154)
			Inhibition of pre-autophagic endosomes	(155)

*Akt, protein kinase B; CaMK, calmodulin kinase; CHO-UT, Chinese hamster ovary line transfected with the human form of the UT receptor; EGFR, epidermal growth factor receptor; ERK, extracellular signal-regulated kinase; FAK, focal adhesion kinase; FoxO3, forkhead box O3; G_i/o_, G_q_, G_13_, G proteins type i/o, q and 13; GSK-3β, glycogen synthase kinase 3β; HB-EGF, heparin-binding EGF-like growth factor; HIF-1, hypoxia inducible factor-1; HUVEC, human umbilical vein endothelial cells; IP_3_, inositol triphosphate; LRP6, low density lipoprotein receptor-related protein 6; JNK, c-Jun N-terminal kinase; MAPK, mitogen-activated protein kinase; MEK, extracellular signal-regulated kinase; MLC, myosin light chain; MMP-2, matrix metalloprotease type 2; NOX, NADPH oxidase; p38MAPK, p38 mitogen-activated protein kinase; PAI-1, plasminogen activator inhibitor-1; PI3K, phosphatidylinositol-3 kinase; PKC, protein kinase C; PKD, protein kinase D; PLC, phospholipase C; PTEN, phosphatase and tensin homolog; Ras, small GTPases; RhoA, Ras homolog gene family, member A; ROCK, rho-associated protein kinase; ROS, reactive oxygen species; SHP-2, src-homology 2-containing tyrosine phosphatase; SMC, smooth muscle cells; TGFβ, transforming growth factor-β; VEGF, vascular endothelial growth factor; N.D., not determined*.

### Effects of UII on the Immune System, Relevant to Chemokine-Like Activity

There are few data concerning the link between urotensinergic and immune systems. Some studies have demonstrated the presence of UT on the surface of selected immune cells, i.e., B and NK lymphocytes, monocytes, and macrophages (145, 156), which infiltrate zones displaying high levels of immunoreactivity for UII (20). UII acts as a chemoattractant for human monocytes (145) and induces the extravasation of plasma in mice (157) and rats (158) (Table 3). Pro-inflammatory signals, such as tumor necrosis factor-α (TNF-α), lipopolysaccharide (LPS), or interferon-γ (IFN-γ), promote the expression of UT (145), while UII induces the secretion of cytokines, such as interleukine-6 (IL-6), in UT transfected human cardiomyocytes and lung adenocarcinoma cells (159, 160). Moreover, UII favors acetyl-coenzyme A acetyltransferase 1 activity in human monocyte (112). On coronary smooth muscle cells or endothelial cells in culture, UII increases the synthesis of inflammatory and pro-thrombotic markers like the plasminogen activator inhibitor-1, the inter-cellular adhesion molecule-1, and the tissue factor through activation of the necrosis factor NF-κB, a pro-inflammatory transcription factor (124, 161). Finally, expression of UT in human leukocytes, especially monocytes and NK cells, is strongly stimulated after exposure to LPS and requires NF-κB (145). In addition, in a mouse model of inflammatory acute liver failure, the expression of UII and UT was significantly increased in liver endothelial cells, and a pretreatment by the UT biased ligand (130) urantide decreased NF-κB activation and inflammatory cytokine (TNF-α, IL-1β, IFN-γ) expression (162).

These data indicate that UII is involved in the immune response and, notably, participates in the production of cytokines and the promotion of immune cell infiltration, suggestive of a chemokine functional activity relayed by the peptide UII, raising a more conserved role in chemotactic attraction of immune cells in pathological situations.

### Chemokine Activity of UII in the Context of Tissue Remodeling and Cancer

Chemotaxis is currently known as the fundamental phenomenon highly conserved from bacteria to eukaryotic cells, implying cell directed migration along an extracellular chemical gradient (163–165), a mechanism essential for a number of physiological and pathological processes including embryogenesis and wiring of the CNS (166, 167), the immune system inflammatory response (168), angiogenesis and cancer cell metastasis, and invasion (165, 169). The “professional” players of chemotaxis, chemokines, are subdivided into C, CC, CXC, and CX3C families, based on the number and spacing of the conserved cysteine residues in their amino termini. Members of the CXC, containing CXCL12 (stromal derived factor-1 or SDF-1) and CC including CCl2 (monocyte chemoattractant protein-1, MCP-1) or CCl5 (regulated upon activation normal T cell, RANTES) chemokine families are known to chemoattract neutrophils, T/B lymphocytes, or natural killer cells and monocytes, macrophages, or T lymphocytes, respectively (170). Through activation of chemotaxis, CXCL12, CCL2, CCL5, or CXCL1 chemokines were shown to stimulate growth, migration/invasion/metastasis as well as angiogenesis and tube formation (171, 172). The CXCL12 and its CXCR4 have long been shown to constitute a promising therapeutic based-system in pre-clinical models and in early clinical trials, but other prototypic chemokines emerge as new potential players in cancer. CCl2 together with its cognate CCR2 play key roles in cancer metastasis by sustaining cancer cell proliferation and survival, stimulating cancer cell migration and invasion, and inducing deleterious inflammation and angiogenesis (173, 174). In addition, various cancer cells produced CCl5 but also expressed CCR1, CCR3, and CCR5, suggesting autocrine/paracrine mechanisms, associated with metalloproteinase activation and invasion (175, 176).

**Figure 3:**
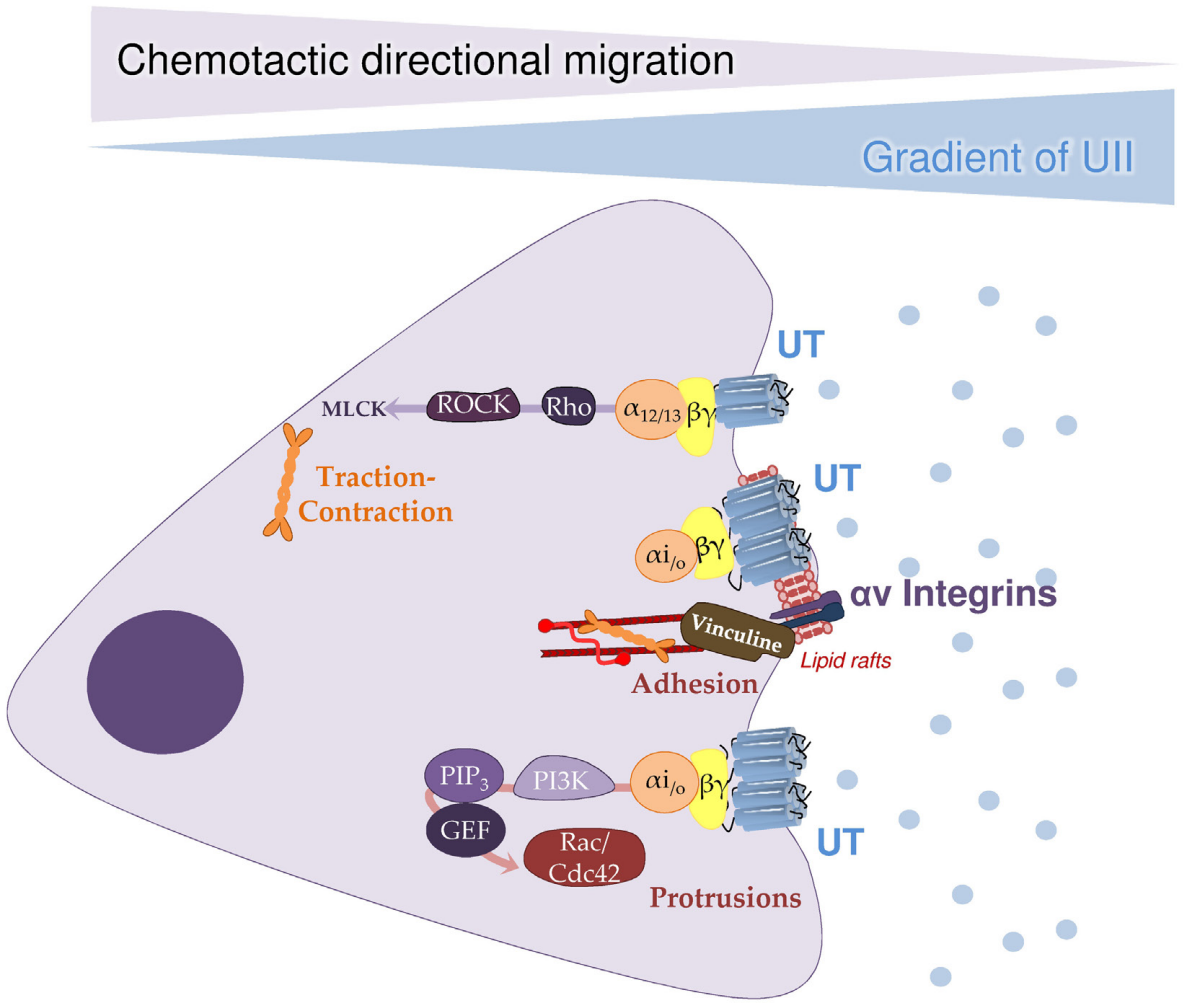
**A hypothetical outline of chemokine signaling cascade relayed by the urotensinergic system inducing cell migration**. Illustration of a pathophysiological situation involving directional migration/invasion of cells expressing UT in response to a urotensin II (UII) gradient concentration. It is proposed that mobile high-affinity UT coupled to both Gαi/o and Gα13 is activated by a low concentration of UII, would promote the formation of protrusions and adhesions at the front of the cell through PI3K/PIP3/GEF/Rac/Cdc42 signaling cascade. At the back of the migrating cell, concomitant activation of G13, likely allows actomyosin contraction *via* the Rho/ROCK/MLCK pathway. To favor cell progression toward the emission source of UII, mobile or engaged UT coupled to Gi/o in lipid rafts may activate proteins responsible for the formation and maturation of focal adhesions composed of αv integrins and vinculin. Together, this pleiotropic UT associated signaling events represents a prototypic chemokine-mediated mechanism shared by P2.58 GPCRs allowing chemotactic migration. Cdc42, cell division control protein 42; GEF, guanine nucleotide exchange factor; MAP1A, microtubule-associated protein 1A; MLCK, myosin light-chain kinase; PI3K, phosphatidylinositol-3 kinase; PIP3, phosphatidylinositol 4,5-trisphosphate; ROCK, rho-associated protein kinase [from Ref. (154)].

Consistent with this, a growing number of independent studies show that UII exerts a stimulatory effect on cell migration (Table 3). The Rho/ROCK signaling pathway appears to play a major role in the effects of UII on the migration of rat fibroblasts (149) and endothelial progenitor cells (146) as well as human monocytes (145). In the latter case, the authors consider UII to be a chemotactic factor that acts on the reorganization of the actin cytoskeleton (Figure 3).

The expression of UT at the endothelial level associated with the pro-migration- and mitotic effects of UII, suggested the involvement of the urotensinergic system in angiogenesis. The first evidence for a proangiogenic effect of UII was obtained by Spinazzi et al., demonstrating that UII leads to the reorganization or tubulogenesis of endothelial cells derived from rat brain microvessels, and stimulates *in vitro* angiogenesis (49). Application of UII in a gelatin implant to the chorioallantoic membrane of chick embryos evokes an increased number of blood vessels (49). Accordingly, studies on human umbilical vein endothelial cells confirmed these data (142, 144, 177) and converge toward chemoattraction of cultured endothelial cells by UII.

The major demonstration of the chemotactic role of the UII/UT system comes from studies on cancer cell lines. The expression of UII and UT is observed in numerous cell lines and tumor samples (Table 3), notably in extracts of adrenal gland tumors, such as adrenocortical carcinomas or pheochromocytomas (178, 179), tumors of the CNS such as glioblastomas or neuroblastomas (44, 180, 181), or tumors of muscular tissue, such as rhabdomyosarcomas (129, 182). To date, few isolated studies have investigated the role of the urotensinergic system in tumorigenesis. For example, UII has been shown to stimulate the proliferation of cells of a pulmonary adenocarcinoma cell line *in vitro* and *in vivo* in a xenograft model in immunodeficient *nude* mice (183). The same team has more recently shown that UII stimulates the release of pro-inflammatory cytokines, such as IL-6, TNF-α, or matrix metalloproteinase-9 and participates in macrophage infiltration of the tumor (160). In human cell lines derived from prostatic or colorectal tumors, application of urantide, Rho pathway inhibitor, or shRNA against UT leads to a decrease in their motility and invasiveness (147, 152). More recently, the expression of UII and UT was also observed in other solid tumors from colon, bladder, and breast (152, 153, 184). The activation of UT with the agonist UII_4–11_ in colon cancer cell lines resulted in stimulation of cell growth whereas the treatment with three biased ligand/antagonists (urantide, UPG83 and UPG85) induced growth inhibition (152). As macrophages have been associated with tumor progression, metastasis, and resistance to treatments (185), these results suggested an important role of UII in chemokine functions associated with tumor development (Table 3).

Definitely, the urotensinergic system appears to be involved in cancer cell motility and invasion. Indeed, our recent work demonstrated in glioma cell lines and in recombinant HEK293 cells, that activation of UT by UII involves a signaling switch through the couplings to Gα13/Rho/ROCK kinases and Gαi/o/PI3K pathways, involved in actin stress fibers, lamellipodia formation and vinculin-stained focal adhesions to initiate directional migration and cell adhesion, sequential mechanisms in tumor invasion (154). This type of mixed couplings were thus proposed for the CCl2/CCR2 system in human bone marrow stem cells in which activation of CCR2 regulates PI3K likely contributing to cell polarity and migration and Rho/ROCK leading to cell retraction (186). Moreover, we provide evidence that UT-induced inhibition of the autophagic process is also a key element in the migration of HEK293 cells expressing UT or CXCR4 as well as U87 glioblastoma cells. Autophagy inhibition after activation of UT or CXCR4 at the leading edge may also locally protect proteins involved in actin remodeling and adhesion assembly, whereas autophagy could remain active at distance from chemotactic GPCRs in order to participate in the disassembly of large focal adhesions (155). Together, the more recent pro-migratory, pro-inflammatory and invasiveness role of the urotensinergic system bring it closer to the chemokine systems, such as CXCL12/CXCR4 or the CCl2/CCR2 pair, widening the therapeutic field of pathologies characterized by cellular migratory events, such development, inflammation, invasion and metastasis.

## Conclusion

In this review, we address the putative UT chemotactic structural and functional definition under an evolutionary angle. According to the postulated evolutionary mechanism, a deletion in TM2 of an ancestral PEP receptor with the P2.59 pattern led by divergence to receptors of the G1 groups with the P2.58 pattern, including UT and chemokine receptors, such as CXCR4. In view of the evolutionary history and chemotaxic properties of UT, we propose that UII/UT may rather be considered as a new chemokine system. Indeed, even if the last decade was mainly devoted to the elucidation of the cardiovascular control by the urotensinergic system, interesting investigations on the pro-inflammatory and pro-migratory properties of UII lead us to stipulate that urotensinergic system must be now considered in a new chemokine therapeutic target in pathological situations involving cell chemoattraction.

## Author Contributions

HC and LD wrote the review and prepared the figures and tables. J-EJ, M-CT, FM, and PG made the bibliography to build the review and constructed the tables and figures. LP and MC participated in the clarification of the UT classification and the establishment of the UT couplings.

## Disclaimer

All appropriate permissions have been obtained from the copyright holders of any work that has been reproduced in this manuscript.

## Conflict of Interest Statement

The authors declare that the research was conducted in the absence of any commercial or financial relationships that could be construed as a potential conflict of interest.

## References

[1] Bern HA Lederis K A reference preparation for the study of active substances in the caudal neurosecretory system of teleosts. J Endocrinol (1969) 45:Sul:xi–xii. 5347394

[2] Conlon JM O’Harte F Smith DD Tonon MC Vaudry H. Isolation and primary structure of urotensin II from the brain of a tetrapod, the frog *Rana ridibunda*. Biochem Biophys Res Commun (1992) 188:578–83. 10.1016/0006-291X(92)91095-8 1445302

[3] Pearson D Shively JE Clark BR Geschwind II Barkley M Nishioka RS Urotensin II: a somatostatin-like peptide in the caudal neurosecretory system of fishes. Proc Natl Acad Sci U S A (1980) 77:5021–4. 10.1073/pnas.77.8.5021 6107911 PMC349982

[4] Ames RS Sarau HM Chambers JK Willette RN Aiyar NV Romanic AM Human urotensin-II is a potent vasoconstrictor and agonist for the orphan receptor GPR14. Nature (1999) 401:282–6. 10.1038/45809 10499587

[5] Coulouarn Y Lihrmann I Jegou S Anouar Y Tostivint H Beauvillain JC Cloning of the cDNA encoding the urotensin II precursor in frog and human reveals intense expression of the urotensin II gene in motoneurons of the spinal cord. Proc Natl Acad Sci U S A (1998) 95:15803–8. 10.1073/pnas.95.26.15803 9861051 PMC28125

[6] González GC Martinez-Padrón M Lederis K Lukowiak K. Distribution and coexistence of urotensin I and urotensin II peptides in the cerebral ganglia of *Aplysia californica*. Peptides (1992) 13:695–703. 10.1016/0196-9781(92)90175-3 1437712

[7] Romanova EV Sasaki K Alexeeva V Vilim FS Jing J Richmond TA Urotensin II in invertebrates: from structure to function in *Aplysia californica*. PLoS One (2012) 7:e48764. 10.1371/journal.pone.0048764 23144960 PMC3493602

[8] Mori M Sugo T Abe M Shimomura Y Kurihara M Kitada C Urotensin II is the endogenous ligand of a G-protein-coupled orphan receptor, SENR (GPR14). Biochem Biophys Res Commun (1999) 265:123–9. 10.1006/bbrc.1999.1640 10548501

[9] Sugo T Murakami Y Shimomura Y Harada M Abe M Ishibashi Y Identification of urotensin II-related peptide as the urotensin II-immunoreactive molecule in the rat brain. Biochem Biophys Res Commun (2003) 310:860–8. 10.1016/j.bbrc.2003.09.102 14550283

[10] Coulouarn Y Jégou S Tostivint H Vaudry H Lihrmann I. Cloning, sequence analysis and tissue distribution of the mouse and rat urotensin II precursors. FEBS Lett (1999) 457:28–32. 10.1016/S0014-5793(99)01003-0 10486557

[11] Elshourbagy NA Douglas SA Shabon U Harrison S Duddy G Sechler JL Molecular and pharmacological characterization of genes encoding urotensin-II peptides and their cognate G-protein-coupled receptors from the mouse and monkey. Br J Pharmacol (2002) 136:9–22. 10.1038/sj.bjp.0704671 11976263 PMC1762106

[12] Russell FD Kearns P Toth I Molenaar P. Urotensin-II-converting enzyme activity of furin and trypsin in human cells in vitro. J Pharmacol Exp Ther (2004) 310:209–14. 10.1124/jpet.104.065425 15007103

[13] Russell FD Meyers D Galbraith AJ Bett N Toth I Kearns P Elevated plasma levels of human urotensin-II immunoreactivity in congestive heart failure. Am J Physiol Heart Circ Physiol (2003) 285:H1576–81. 10.1152/ajpheart.00217.2003 12791592

[14] Sugo T Mori M. Another ligand fishing for G protein-coupled receptor 14. Discovery of urotensin II-related peptide in the rat brain. Peptides (2008) 29:809–12. 10.1016/j.peptides.2007.06.005 17628210

[15] Nobata S Donald JA Balment RJ Takei Y. Potent cardiovascular effects of homologous urotensin II (UII)-related peptide and UII in unanesthetized eels after peripheral and central injections. Am J Physiol Regul Integr Comp Physiol (2011) 300:R437–46. 10.1152/ajpregu.00629.2010 21123764

[16] Quan FB Bougerol M Rigour F Kenigfest NB Tostivint H. Characterization of the true ortholog of the urotensin II-related peptide (URP) gene in teleosts. Gen Comp Endocrinol (2012) 177:205–12. 10.1016/j.ygcen.2012.02.018 22433941

[17] Tostivint H Joly L Lihrmann I Parmentier C Lebon A Morisson M Comparative genomics provides evidence for close evolutionary relationships between the urotensin II and somatostatin gene families. Proc Natl Acad Sci U S A (2006) 103:2237–42. 10.1073/pnas.0510700103 16467151 PMC1413727

[18] Douglas SA Tayara L Ohlstein EH Halawa N Giaid A. Congestive heart failure and expression of myocardial urotensin II. Lancet (2002) 359:1990–7. 10.1016/S0140-6736(02)08831-1 12076554

[19] Dschietzig T Bartsch C Pregla R Zurbrügg HR Armbruster FP Richter C Plasma levels and cardiovascular gene expression of urotensin-II in human heart failure. Regul Pept (2002) 110:33–8. 10.1016/S0167-0115(02)00158-1 12468107

[20] Maguire JJ Kuc RE Wiley KE Kleinz MJ Davenport AP. Cellular distribution of immunoreactive urotensin-II in human tissues with evidence of increased expression in atherosclerosis and a greater constrictor response of small compared to large coronary arteries. Peptides (2004) 25:1767–74. 10.1016/j.peptides.2004.01.028 15476944

[21] Matsushita M Shichiri M Imai T Iwashina M Tanaka H Takasu N Co-expression of urotensin II and its receptor (GPR14) in human cardiovascular and renal tissues. J Hypertens (2001) 19:2185–90. 10.1097/00004872-200112000-00011 11725162

[22] Dubessy C Cartier D Lectez B Bucharles C Chartrel N Montero-Hadjadje M Characterization of urotensin II, distribution of urotensin II, urotensin II-related peptide and UT receptor mRNAs in mouse: evidence of urotensin II at the neuromuscular junction. J Neurochem (2008) 107:361–74. 10.1111/j.1471-4159.2008.05624.x 18710417

[23] Totsune K Takahashi K Arihara Z Sone M Satoh F Ito S Role of urotensin II in patients on dialysis. Lancet (2001) 358:810–1. 10.1016/S0140-6736(01)06002-0 11564491

[24] Totsune K Takahashi K Arihara Z Sone M Ito S Murakami O Increased plasma urotensin II levels in patients with diabetes mellitus. Clin Sci (Lond) (2003) 104:1–5. 10.1042/cs1040001 12519081

[25] Shenouda A Douglas SA Ohlstein EH Giaid A. Localization of urotensin-II immunoreactivity in normal human kidneys and renal carcinoma. J Histochem Cytochem (2002) 50:885–9. 10.1177/002215540205000702 12070267

[26] Silvestre RA Rodríguez-Gallardo J Egido EM Marco J Inhibition of insulin release by urotensin II – a study on the perfused rat pancreas. Horm Metab Res (2001) 33:379–81. 10.1055/s-2001-15414 11456289

[27] Hirose T Takahashi K Mori N Nakayama T Kikuya M Ohkubo T Increased expression of urotensin II, urotensin II-related peptide and urotensin II receptor mRNAs in the cardiovascular organs of hypertensive rats: comparison with endothelin-1. Peptides (2009) 30:1124–9. 10.1016/j.peptides.2009.02.009 19463745

[28] Nakayama T Hirose T Totsune K Mori N Maruyama Y Maejima T Increased gene expression of urotensin II-related peptide in the hearts of rats with congestive heart failure. Peptides (2008) 29:801–8. 10.1016/j.peptides.2007.12.018 18314225

[29] Kristof AS You Z Han Y-S Giaid A. Protein expression of urotensin II, urotensin-related peptide and their receptor in the lungs of patients with lymphangioleiomyomatosis. Peptides (2010) 31:1511–6. 10.1016/j.peptides.2010.04.017 20433884 PMC2905484

[30] Wang H Dong K Xue X Feng P Wang X. Elevated expression of urotensin II and its receptor in diethylnitrosamine-mediated precancerous lesions in rat liver. Peptides (2011) 32:382–7. 10.1016/j.peptides.2010.10.032 21056072

[31] Dun SL Brailoiu GC Yang J Chang JK Dun NJ. Urotensin II-immunoreactivity in the brainstem and spinal cord of the rat. Neurosci Lett (2001) 305:9–12. 10.1016/S0304-3940(01)01804-3 11356295

[32] Egginger J-G Camus A Calas A Urotensin-II expression in the mouse spinal cord. J Chem Neuroanat (2006) 31:146–54. 10.1016/j.jchemneu.2005.10.004 16361078

[33] Egginger J-G Calas A. A novel hypothalamic neuroendocrine peptide: URP (urotensin-II-related peptide)? C R Biol (2005) 328:724–31. 10.1016/j.crvi.2005.06.002 16125650

[34] Tal M Ammar DA Karpuj M Krizhanovsky V Naim M Thompson DA. A novel putative neuropeptide receptor expressed in neural tissue, including sensory epithelia. Biochem Biophys Res Commun (1995) 209:752–9. 10.1006/bbrc.1995.1563 7733947

[35] Marchese A Heiber M Nguyen T Heng HHQ Saldivia VR Cheng R Cloning and chromosomal mapping of three novel genes, GPR9, GPR10, and GPR14, encoding receptors related to interleukin 8, neuropeptide Y, and somatostatin receptors. Genomics (1995) 29:335–44. 10.1006/geno.1995.9996 8666380

[36] Liu Q Pong SS Zeng Z Zhang Q Howard AD Williams DL Identification of urotensin II as the endogenous ligand for the orphan G-protein-coupled receptor GPR14. Biochem Biophys Res Commun (1999) 266:174–8. 10.1006/bbrc.1999.1796 10581185

[37] Nothacker H-P Wang Z McNeill AM Saito Y Merten S O’Dowd B Identification of the natural ligand of an orphan G-protein-coupled receptor involved in the regulation of vasoconstriction. Nat Cell Biol (1999) 1:383–5. 10.1038/14081 10559967

[38] Gartlon J Parker F Harrison DC Douglas SA Ashmeade TE Riley GJ Central effects of urotensin-II following ICV administration in rats. Psychopharmacology (Berl) (2001) 155:426–33. 10.1007/s002130100715 11441433

[39] Gong H Wang Y-X Zhu Y-Z Wang W-W Wang M-J Yao T Cellular distribution of GPR14 and the positive inotropic role of urotensin II in the myocardium in adult rat. J Appl Physiol (2004) 97:2228–35. 10.1152/japplphysiol.00540.2004 15273242

[40] Leonard AD Thompson JP Hutchinson EL Young SP McDonald J Swanevelder J Urotensin II receptor expression in human right atrium and aorta: effects of ischaemic heart disease. Br J Anaesth (2009) 102:477–84. 10.1093/bja/aep011 19258379

[41] Maguire JJ Kuc RE Davenport AP. Orphan-receptor ligand human urotensin II: receptor localization in human tissues and comparison of vasoconstrictor responses with endothelin-1. Br J Pharmacol (2000) 131:441–6. 10.1038/sj.bjp.0703601 11015293 PMC1572358

[42] Maguire JJ Kuc RE Kleinz MJ Davenport AP. Immunocytochemical localization of the urotensin-II receptor, UT, to rat and human tissues: relevance to function. Peptides (2008) 29:735–42. 10.1016/j.peptides.2007.08.021 17905478

[43] Mori N Hirose T Nakayama T Ito O Kanazawa M Imai Y Increased expression of urotensin II-related peptide and its receptor in kidney with hypertension or renal failure. Peptides (2009) 30:400–8. 10.1016/j.peptides.2008.09.021 18955095

[44] Nguyen T-TM Létourneau M Chatenet D Fournier A. Presence of urotensin-II receptors at the cell nucleus: specific tissue distribution and hypoxia-induced modulation. Int J Biochem Cell Biol (2012) 44:639–47. 10.1016/j.biocel.2011.12.022 22245063

[45] Song W McDonald J Camarda V Calo G Guerrini R Marzola E Cell and tissue responses of a range of Urotensin II analogs at cloned and native urotensin II receptors. Evidence for coupling promiscuity. Naunyn Schmiedebergs Arch Pharmacol (2006) 373:148–57. 10.1007/s00210-006-0057-2 16596397

[46] Tian L Li C Qi J Fu P Yu X Li X Diabetes-induced upregulation of urotensin II and its receptor plays an important role in TGF-beta1-mediated renal fibrosis and dysfunction. Am J Physiol Endocrinol Metab (2008) 295:E1234–42. 10.1152/ajpendo.90672.2008 18796544

[47] Langham RG Kelly DJ Gow RM Zhang Y Dowling JK Thomson NM Increased expression of urotensin II and urotensin II receptor in human diabetic nephropathy. Am J Kidney Dis (2004) 44:826–31. 10.1016/S0272-6386(04)01130-8 15492948

[48] Jégou S Cartier D Dubessy C Gonzalez BJ Chatenet D Tostivint H Localization of the urotensin II receptor in the rat central nervous system. J Comp Neurol (2006) 495:21–36. 10.1002/cne.20845 16432902

[49] Spinazzi R Albertin G Nico B Guidolin D Di Liddo R Rossi GP Urotensin-II and its receptor (UT-R) are expressed in rat brain endothelial cells, and urotensin-II via UT-R stimulates angiogenesis in vivo and in vitro. Int J Mol Med (2006) 18:1107–12. 10.3892/ijmm.18.6.1107 17089015

[50] Lin Y Tsuchihashi T Matsumura K Fukuhara M Ohya Y Fujii K Central cardiovascular action of urotensin II in spontaneously hypertensive rats. Hypertens (2003) 26:839–45. 10.1291/hypres.26.839 14621188

[51] Castel H Diallo M Chatenet D Leprince J Desrues L Schouft M-T Biochemical and functional characterization of high-affinity urotensin II receptors in rat cortical astrocytes. J Neurochem (2006) 99:582–95. 10.1111/j.1471-4159.2006.04130.x 16942596

[52] Jani PP Narayan H Ng LL. The differential extraction and immunoluminometric assay of urotensin II and urotensin-related peptide in heart failure. Peptides (2013) 40:72–6. 10.1016/j.peptides.2012.12.014 23270674

[53] Ng LL Loke I O’Brien RJ Squire IB Davies JE Plasma urotensin in human systolic heart failure. Circulation (2002) 106:2877–80. 10.1161/01.CIR.0000044388.19119.02 12460864

[54] Kemp W Roberts S Krum H. Increased circulating urotensin II in cirrhosis: potential implications in liver disease. Peptides (2008) 29:868–72. 10.1016/j.peptides.2007.08.020 17913301

[55] Heller J Schepke M Neef M Woitas R Rabe C Sauerbruch T. Increased urotensin II plasma levels in patients with cirrhosis and portal hypertension. J Hepatol (2002) 37:767–72. 10.1016/S0168-8278(02)00295-7 12445417

[56] Pawar R Kemp W Roberts S Krum H Yandle T Hardikar W. Urotensin II levels are an important marker for the severity of portal hypertension in children. J Pediatr Gastroenterol Nutr (2011) 53:88–92. 10.1097/MPG.0b013e3182153900 21694541

[57] Suguro T Watanabe T Ban Y Kodate S Misaki A Hirano T Increased human urotensin II levels are correlated with carotid atherosclerosis in essential hypertension. Am J Hypertens (2007) 20:211–7. 10.1016/j.amjhyper.2006.08.001 17261470

[58] Pakala R Role of urotensin II in atherosclerotic cardiovascular diseases. Cardiovasc Revasc Med (2008) 9:166–78. 10.1016/j.carrev.2008.02.001 18606380

[59] Tzanidis A Hannan RD Thomas WG Onan D Autelitano DJ See F Direct actions of urotensin II on the heart: implications for cardiac fibrosis and hypertrophy. Circ Res (2003) 93:246–53. 10.1161/01.RES.0000084382.64418.BC 12842917

[60] Bockaert J Pin JP. Molecular tinkering of G protein-coupled receptors: an evolutionary success. EMBO J (1999) 18:1723–9. 10.1093/emboj/18.7.1723 10202136 PMC1171258

[61] Attwood TK Findlay JBC. Fingerprinting G-protein-coupled receptors. Protein Eng (1994) 7:195–203. 10.1093/protein/7.2.195 8170923

[62] Fenalti G Giguere PM Katritch V Huang X-P Thompson AA Cherezov V Molecular control of d-opioid receptor signalling. Nature (2014) 506:191–6. 10.1038/nature12944 24413399 PMC3931418

[63] Manglik A Kruse AC Kobilka TS Thian FS Mathiesen JM Sunahara RK Crystal structure of the µ-opioid receptor bound to a morphinan antagonist. Nature (2012) 485:321–6. 10.1038/nature10954 22437502 PMC3523197

[64] Fredriksson R Lagerström MC Lundin L-G Schiöth HB. The G-protein-coupled receptors in the human genome form five main families. Phylogenetic analysis, paralogon groups, and fingerprints. Mol Pharmacol (2003) 63:1256–72. 10.1124/mol.63.6.1256 12761335

[65] Ballesteros JA Weinstein H Integrated methods for the construction of three-dimensional models and computational probing of structure-function relations in G protein-coupled receptors. Methods Neurosci (1995) 25:366–428. 10.1016/S1043-9471(05)80049-7

[66] Tautermann CS. GPCR structures in drug design, emerging opportunities with new structures. Bioorg Med Chem Lett (2014) 24:4073–9. 10.1016/j.bmcl.2014.07.009 25086683

[67] Lin H Sassano MF Roth BL Shoichet BK. A pharmacological organization of G protein-coupled receptors. Nat Methods (2013) 10:140–6. 10.1038/nmeth.2324 23291723 PMC3560304

[68] Onan D Hannan RD Thomas WG Urotensin II: the old kid in town. Trends Endocrinol Metab (2004) 15:175–82. 10.1016/j.tem.2004.03.007 15109617

[69] Chatenet D Nguyen T-TM Létourneau M Fournier A Update on the urotensinergic system: new trends in receptor localization, activation, and drug design. Front Endocrinol (2012) 3:174 10.3389/fendo.2012.00174 PMC3533682 23293631

[70] Proulx CD Holleran BJ Boucard AA Escher E Guillemette G Leduc R Mutational analysis of the conserved Asp2.50 and ERY motif reveals signaling bias of the urotensin II receptor. Mol Pharmacol (2008) 74:552–61. 10.1124/mol.108.045054 18509066

[71] Oakley RH Laporte SA Holt JA Barak LS Caron MG Molecular determinants underlying the formation of stable intracellular G protein-coupled receptor-β-arrestin complexes after receptor endocytosis*. J Biol Chem (2001) 276:19452–60. 10.1074/jbc.M101450200 11279203

[72] Proulx CD Holleran BJ Lavigne P Escher E Guillemette G Leduc R Biological properties and functional determinants of the urotensin II receptor. Peptides (2008) 29:691–9. 10.1016/j.peptides.2007.10.027 18155322

[73] Chen Y Shang Y Xu D Multi-dimensional scaling and MODELLER-based evolutionary algorithms for protein model refinement. Proc Congr Evol Comput (2014) 2014:1038–45. 10.1109/CEC.2014.6900443 25844403 PMC4380876

[74] Hout MC Papesh MH Goldinger SD. Multidimensional scaling. Wiley Interdiscip Rev Cogn Sci (2013) 4:93–103. 10.1002/wcs.1203 23359318 PMC3555222

[75] Chabbert M Castel H Pele J Deville J Legendre R Rodien P. Evolution of class A G-protein-coupled receptors: implications for molecular modeling. Curr Med Chem (2012) 19:1110–8. 10.2174/092986712799320600 22300045

[76] Pelé J Abdi H Moreau M Thybert D Chabbert M. Multidimensional scaling reveals the main evolutionary pathways of class A G-protein-coupled receptors. PLoS One (2011) 6:e19094. 10.1371/journal.pone.0019094 21544207 PMC3081337

[77] Devillé J Rey J Chabbert M. An indel in transmembrane helix 2 helps to trace the molecular evolution of class A G-protein-coupled receptors. J Mol Evol (2009) 68:475–89. 10.1007/s00239-009-9214-9 19357801

[78] Visiers I Ballesteros JA Weinstein H Three-dimensional representations of G protein-coupled receptor structures and mechanisms. Methods Enzymol (2002) 343:329–71. 10.1016/S0076-6879(02)43145-X 11665578

[79] Palczewski K Kumasaka T Hori T Behnke CA Motoshima H Fox BA Crystal structure of rhodopsin: a G protein-coupled receptor. Science (2000) 289:739–45. 10.1126/science.289.5480.739 10926528

[80] Rasmussen SGF Choi H-J Rosenbaum DM Kobilka TS Thian FS Edwards PC Crystal structure of the human β2 adrenergic G-protein-coupled receptor. Nature (2007) 450:383–7. 10.1038/nature06325 17952055

[81] Wu B Chien EYT Mol CD Fenalti G Liu W Katritch V Structures of the CXCR4 chemokine GPCR with small-molecule and cyclic peptide antagonists. Science (2010) 330:1066–71. 10.1126/science.1194396 20929726 PMC3074590

[82] Yohannan S Faham S Yang D Whitelegge JP Bowie JU. The evolution of transmembrane helix kinks and the structural diversity of G protein-coupled receptors. Proc Natl Acad Sci U S A (2004) 101:959–63. 10.1073/pnas.0306077101 14732697 PMC327124

[83] Chatenet D Dubessy C Leprince J Boularan C Carlier L Ségalas-Milazzo I Structure-activity relationships and structural conformation of a novel urotensin II-related peptide. Peptides (2004) 25:1819–30. 10.1016/j.peptides.2004.04.019 15476952

[84] Labarrère P Chatenet D Leprince J Marionneau C Loirand G Tonon M-C Structure-activity relationships of human urotensin II and related analogues on rat aortic ring contraction. J Enzyme Inhib Med Chem (2003) 18:77–88. 10.1080/1475636031000093507 12943190

[85] Opgaard OS Nothacker H-P Ehlert FJ Krause DN. Human urotensin II mediates vasoconstriction via an increase in inositol phosphates. Eur J Pharmacol (2000) 406:265–71. 10.1016/S0014-2999(00)00672-5 11020490

[86] Camarda V Guerrini R Kostenis E Rizzi A Calò G Hattenberger A A new ligand for the urotensin II receptor. Br J Pharmacol (2002) 137:311–4. 10.1038/sj.bjp.0704895 12237249 PMC1573506

[87] MacLean MR Alexander D Stirrat A Gallagher M Douglas SA Ohlstein EH Contractile responses to human urotensin-II in rat and human pulmonary arteries: effect of endothelial factors and chronic hypoxia in the rat. Br J Pharmacol (2000) 130:201–4. 10.1038/sj.bjp.0703314 10807654 PMC1572074

[88] Russell FD Molenaar P O’Brien DM. Cardiostimulant effects of urotensin-II in human heart in vitro. Br J Pharmacol (2001) 132:5–9. 10.1038/sj.bjp.0703811 11156554 PMC1572555

[89] Gibson A. Complex effects of *Gillichthys* urotensin II on rat aortic strips. Br J Pharmacol (1987) 91:205–12. 10.1111/j.1476-5381.1987.tb09000.x 2885055 PMC1853480

[90] Gibson A Conyers S Bern HA. The influence of urotensin II on calcium flux in rat aorta. J Pharm Pharmacol (1988) 40:893–5. 10.1111/j.2042-7158.1988.tb06298.x 2907588

[91] Itoh H Itoh Y Rivier J Lederis K. Contraction of major artery segments of rat by fish neuropeptide urotensin II. Am J Physiol (1987) 252:R361–6. 3812773 10.1152/ajpregu.1987.252.2.R361

[92] Rossowski WJ Cheng B-L Taylor JE Datta R Coy DH. Human urotensin II-induced aorta ring contractions are mediated by protein kinase C, tyrosine kinases and Rho-kinase: inhibition by somatostatin receptor antagonists. Eur J Pharmacol (2002) 438:159–70. 10.1016/S0014-2999(02)01341-9 11909607

[93] Russell FD Molenaar P. Investigation of signaling pathways that mediate the inotropic effect of urotensin-II in human heart. Cardiovasc Res (2004) 63:673–81. 10.1016/j.cardiores.2004.05.009 15306223

[94] Tasaki K Hori M Ozaki H Karaki H Wakabayashi I. Mechanism of human urotensin II-induced contraction in rat aorta. J Pharmacol Sci (2004) 94:376–83. 10.1254/jphs.94.376 15107577

[95] Sauzeau V Le Mellionnec E Bertoglio J Scalbert E Pacaud P Loirand G Human urotensin II-induced contraction and arterial smooth muscle cell proliferation are mediated by RhoA and Rho-kinase. Circ Res (2001) 88:1102–4. 10.1161/hh1101.092034 11397774

[96] Abdelrahman AM Pang CCY. Involvement of the nitric oxide/l-arginine and sympathetic nervous systems on the vasodepressor action of human urotensin II in anesthetized rats. Life Sci (2002) 71:819–25. 10.1016/S0024-3205(02)01743-5 12074941

[97] Gardiner SM March JE Kemp PA Davenport AP Bennett T. Depressor and regionally-selective vasodilator effects of human and rat urotensin II in conscious rats. Br J Pharmacol (2001) 132:1625–9. 10.1038/sj.bjp.0704051 11309232 PMC1572749

[98] Hassan GS Chouiali F Saito T Hu F Douglas SA Ao Z Effect of human urotensin-II infusion on hemodynamics and cardiac function. Can J Physiol Pharmacol (2003) 81:125–8. 10.1139/y03-004 12710525

[99] Kompa AR Thomas WG See F Tzanidis A Hannan RD Krum H. Cardiovascular role of urotensin II: effect of chronic infusion in the rat. Peptides (2004) 25:1783–8. 10.1016/j.peptides.2004.03.029 15476946

[100] Zhu YZ Wang ZJ Zhu YC Zhang L Oakley RME Chung CW Urotensin II causes fatal circulatory collapse in anesthesized monkeys in vivo: a “vasoconstrictor” with a unique hemodynamic profile. Am J Physiol Heart Circ Physiol (2004) 286:H830–6. 10.1152/ajpheart.00406.2003 14615276

[101] Böhm F Pernow J. Urotensin II evokes potent vasoconstriction in humans in vivo. Br J Pharmacol (2002) 135:25–7. 10.1038/sj.bjp.0704448 11786476 PMC1573112

[102] Affolter JT Newby DE Wilkinson IB Winter MJ Balment RJ Webb DJ. No effect on central or peripheral blood pressure of systemic urotensin II infusion in humans. Br J Clin Pharmacol (2002) 54:617–21. 10.1046/j.1365-2125.2002.t01-1-01704.x 12492609 PMC1874506

[103] Cheriyan J Burton TJ Bradley TJ Wallace SML Mäki-Petäjä KM Mackenzie IS The effects of urotensin II and urantide on forearm blood flow and systemic haemodynamics in humans. Br J Clin Pharmacol (2009) 68:518–23. 10.1111/j.1365-2125.2009.03475.x 19843055 PMC2780277

[104] Wilkinson IB Affolter JT de Haas SL Pellegrini MP Boyd J Winter MJ High plasma concentrations of human urotensin II do not alter local or systemic hemodynamics in man. Cardiovasc Res (2002) 53:341–7. 10.1016/S0008-6363(01)00485-0 11827684

[105] Lim M Honisett S Sparkes CD Komesaroff P Kompa A Krum H. Differential effect of urotensin II on vascular tone in normal subjects and patients with chronic heart failure. Circulation (2004) 109:1212–4. 10.1161/01.CIR.0000121326.69153.98 15007012

[106] Sondermeijer B Kompa A Komesaroff P Krum H. Effect of exogenous urotensin-II on vascular tone in skin microcirculation of patients with essential hypertension. Am J Hypertens (2005) 18:1195–9. 10.1016/j.amjhyper.2005.03.748 16182109

[107] Zomer E de Ridder I Kompa A Komesaroff P Gilbert R Krum H. Effect of urotensin II on skin microvessel tone in diabetic patients without heart failure or essential hypertension. Clin Exp Pharmacol Physiol (2008) 35:1147–50. 10.1111/j.1440-1681.2008.04960.x 18505448

[108] Lapp H Boerrigter G Costello-Boerrigter LC Jaekel K Scheffold T Krakau I Elevated plasma human urotensin-II-like immunoreactivity in ischemic cardiomyopathy. Int J Cardiol (2004) 94:93–7. 10.1016/j.ijcard.2003.05.008 14996481

[109] Hassan GS Douglas SA Ohlstein EH Giaid A Expression of urotensin-II in human coronary atherosclerosis. Peptides (2005) 26:2464–72. 10.1016/j.peptides.2005.05.028 16026900

[110] Watanabe T Pakala R Katagiri T Benedict CR. Synergistic effect of urotensin II with serotonin on vascular smooth muscle cell proliferation. J Hypertens (2001) 19:2191–6. 10.1097/00004872-200112000-00012 11725163

[111] Shiraishi Y Watanabe T Suguro T Nagashima M Kato R Hongo S Chronic urotensin II infusion enhances macrophage foam cell formation and atherosclerosis in apolipoprotein E-knockout mice. J Hypertens (2008) 26:1955–65. 10.1097/HJH.0b013e32830b61d8 18806619

[112] Watanabe T Suguro T Kanome T Sakamoto Y-I Kodate S Hagiwara T Human urotensin II accelerates foam cell formation in human monocyte-derived macrophages. Hypertension (2005) 1979(46):738–44. 10.1161/01.HYP.0000184226.99196.b5 16172428

[113] Bousette N Giaid A. Urotensin-II and cardiovascular diseases. Curr Hypertens Rep (2006) 8:479–83. 10.1007/s11906-006-0026-7 17139807

[114] Rakowski E Hassan GS Dhanak D Ohlstein EH Douglas SA Giaid A. A role for urotensin II in restenosis following balloon angioplasty: use of a selective UT receptor blocker. J Mol Cell Cardiol (2005) 39:785–91. 10.1016/j.yjmcc.2005.07.002 16171813

[115] Pehlivan Y Dokuyucu R Demir T Kaplan DS Koc I Orkmez M Palosuran treatment effective as bosentan in the treatment model of pulmonary arterial hypertension. Inflammation (2014) 37:1280–8. 10.1007/s10753-014-9855-8 24604341

[116] Zhao J Xie L-D Song C-J Mao X-X Yu H-R Yu Q-X Urantide improves atherosclerosis by controlling C-reactive protein, monocyte chemotactic protein-1 and transforming growth factor-β expression in rats. Exp Ther Med (2014) 7:1647–52. 10.3892/etm.2014.1654 24926360 PMC4043621

[117] You Z Genest J Barrette P-O Hafiane A Behm DJ D’Orleans-Juste P Genetic and pharmacological manipulation of urotensin II ameliorate the metabolic and atherosclerosis sequalae in mice. Arterioscler Thromb Vasc Biol (2012) 32:1809–16. 10.1161/ATVBAHA.112.252973 22723440

[118] Ziltener P Mueller C Haenig B Scherz MW Nayler O. Urotensin II mediates Erk1/2 phosphorylation and proliferation in GPR14-transfected cell lines. J Recept Signal Transduct Res (2002) 22:155–68. 10.1081/RRS-120014593 12503613

[119] Matsushita M Shichiri M Fukai N Ozawa N Yoshimoto T Takasu N Urotensin II is an autocrine/paracrine growth factor for the porcine renal epithelial cell line, LLCPK1. Endocrinology (2003) 144:1825–31. 10.1210/en.2003-0029 12697688

[120] Zhang W-X Liang Y-F Wang X-M Nie Y Chong L Lin L Urotensin upregulates transforming growth factor-β1 expression of asthma airway through ERK-dependent pathway. Mol Cell Biochem (2012) 364:291–8. 10.1007/s11010-012-1229-7 22270542

[121] Chen Y-L Liu J-C Loh S-H Chen C-H Hong C-Y Chen J-J Involvement of reactive oxygen species in urotensin II-induced proliferation of cardiac fibroblasts. Eur J Pharmacol (2008) 593:24–9. 10.1016/j.ejphar.2008.07.025 18671962

[122] Sue Y-M Chen C-H Hsu Y-H Hou C-C Cheng C-Y Chen Y-C Urotensin II induces transactivation of the epidermal growth factor receptor via transient oxidation of SHP-2 in the rat renal tubular cell line NRK-52E. Growth Factors (2009) 27:155–62. 10.1080/08977190902879866 19326266

[123] Tsai C-S Loh S-H Liu J-C Lin J-W Chen Y-L Chen C-H Urotensin II-induced endothelin-1 expression and cell proliferation via epidermal growth factor receptor transactivation in rat aortic smooth muscle cells. Atherosclerosis (2009) 206:86–94. 10.1016/j.atherosclerosis.2009.02.013 19269634

[124] Djordjevic T BelAiba RS Bonello S Pfeilschifter J Hess J Görlach A. Human urotensin II is a novel activator of NADPH oxidase in human pulmonary artery smooth muscle cells. Arterioscler Thromb Vasc Biol (2005) 25:519–25. 10.1161/01.ATV.0000154279.98244.eb 15618545

[125] Liu J-C Chen C-H Chen J-J Cheng T-H. Urotensin II induces rat cardiomyocyte hypertrophy via the transient oxidization of Src homology 2-containing tyrosine phosphatase and transactivation of epidermal growth factor receptor. Mol Pharmacol (2009) 76:1186–95. 10.1124/mol.109.058297 19755521

[126] Onan D Pipolo L Yang E Hannan RD Thomas WG Urotensin II promotes hypertrophy of cardiac myocytes via mitogen-activated protein kinases. Mol Endocrinol (2004) 18:2344–54. 10.1210/me.2003-0309 15205471

[127] Desrues L Lefebvre T Diallo M Gandolfo P Leprince J Chatenet D Effect of GABA A receptor activation on UT-coupled signaling pathways in rat cortical astrocytes. Peptides (2008) 29:727–34. 10.1016/j.peptides.2008.01.024 18355946

[128] Jarry M Diallo M Lecointre C Desrues L Tokay T Chatenet D The vasoactive peptides urotensin II and urotensin II-related peptide regulate astrocyte activity through common and distinct mechanisms: involvement in cell proliferation. Biochem J (2010) 428:113–24. 10.1042/BJ20090867 20192922

[129] Douglas SA Naselsky D Ao Z Disa J Herold CL Lynch F Identification and pharmacological characterization of native, functional human urotensin-II receptors in rhabdomyosarcoma cell lines. Br J Pharmacol (2004) 142:921–32. 10.1038/sj.bjp.0705743 15210573 PMC1575108

[130] Brulé C Perzo N Joubert J-E Sainsily X Leduc R Castel H Biased signaling regulates the pleiotropic effects of the urotensin II receptor to modulate its cellular behaviors. FASEB J (2014) 28:5148–62. 10.1096/fj.14-249771 25183668

[131] Chen Y Zhao M Liu X Yao W Yang J Zhang Z Urotensin II receptor in the rat airway smooth muscle and its effect on the rat airway smooth muscle cells proliferation. Chin Med Sci J (2001) 16:231–5. 12903763

[132] Iglewski M Grant SR. Urotensin II-induced signaling involved in proliferation of vascular smooth muscle cells. Vasc Health Risk Manag (2010) 6:723–34. 10.2147/VHRM.S11129 20859543 PMC2941785

[133] Xu S Wen H Jiang H. Urotensin II promotes the proliferation of endothelial progenitor cells through p38 and p44/42 MAPK activation. Mol Med Rep (2012) 6:197–200. 10.3892/mmr.2012.899 22552405

[134] Diebold I Petry A Burger M Hess J Görlach A. NOX4 mediates activation of FoxO3a and matrix metalloproteinase-2 expression by urotensin-II. Mol Biol Cell (2011) 22:4424–34. 10.1091/mbc.E10-12-0971 21965295 PMC3216667

[135] Song N Ding W Chu S Zhao J Dong X Di B Urotensin II stimulates vascular endothelial growth factor secretion from adventitial fibroblasts in synergy with angiotensin II. Circ J (2012) 76:1267–73. 10.1253/circj.CJ-11-0870 22382381

[136] Lee S-J Jung YH Oh SY Yun SP Han HJ. Melatonin enhances the human mesenchymal stem cells motility via melatonin receptor 2 coupling with Gαq in skin wound healing. J Pineal Res (2014) 57:393–407. 10.1111/jpi.12179 25250716

[137] Chen G Wang W Meng S Zhang L Wang W Jiang Z CXC chemokine CXCL12 and its receptor CXCR4 in tree shrews (*Tupaia belangeri*): structure, expression and function. PLoS One (2014) 9:e98231 10.1371/journal.pone.0098231 24858548 PMC4032326

[138] Esposito G Perrino C Cannavo A Schiattarella GG Borgia F Sannino A EGFR trans-activation by urotensin II receptor is mediated by β-arrestin recruitment and confers cardioprotection in pressure overload-induced cardiac hypertrophy. Basic Res Cardiol (2011) 106:577–89. 10.1007/s00395-011-0163-2 21369867

[139] Chen L Jin L Zhou N. An update of novel screening methods for GPCR in drug discovery. Expert Opin Drug Discov (2012) 7:791–806. 10.1517/17460441.2012.699036 22716301

[140] Gruson D Ginion A Decroly N Lause P Vanoverschelde JL Ketelslegers JM Urotensin II induction of adult cardiomyocytes hypertrophy involves the Akt/GSK-3beta signaling pathway. Peptides (2010) 31:1326–33. 10.1016/j.peptides.2010.04.009 20416349

[141] Chao H-H Sung L-C Chen C-H Liu J-C Chen J-J Cheng T-H Lycopene inhibits urotensin-II-induced cardiomyocyte hypertrophy in neonatal rat cardiomyocytes. Evid Based Complement Alternat Med (2014) 2014:724670 10.1155/2014/724670 24971153 PMC4058208

[142] Guidolin D Albertin G Ribatti D Urotensin-II as an angiogenic factor. Peptides (2010) 31:1219–24. 10.1016/j.peptides.2010.03.022 20346384

[143] Albertin G Guidolin D Sorato E Oselladore B Tortorella C Ribatti D. Urotensin-II-stimulated expression of pro-angiogenic factors in human vascular endothelial cells. Regul Pept (2011) 172:16–22. 10.1016/j.regpep.2011.08.001 21871928

[144] Diebold I Petry A Sabrane K Djordjevic T Hess J Görlach A. The HIF1 target gene NOX2 promotes angiogenesis through urotensin-II. J Cell Sci (2012) 125:956–64. 10.1242/jcs.094060 22399808

[145] Segain J-P Rolli-Derkinderen M Gervois N Raingeard de la Blétière D Loirand G Pacaud P Urotensin II is a new chemotactic factor for UT receptor-expressing monocytes. J Immunol (2007) 179:901–9. 10.4049/jimmunol.179.2.901 17617581

[146] Xu S Jiang H Wu B Yang J Chen S. Urotensin II induces migration of endothelial progenitor cells via activation of the RhoA/Rho kinase pathway. Tohoku J Exp Med (2009) 219:283–8. 10.1620/tjem.219.283 19966526

[147] Grieco P Franco R Bozzuto G Toccacieli L Sgambato A Marra M Urotensin II receptor predicts the clinical outcome of prostate cancer patients and is involved in the regulation of motility of prostate adenocarcinoma cells. J Cell Biochem (2011) 112:341–53. 10.1002/jcb.22933 21080343

[148] Matsusaka S Wakabayashi I. Enhancement of vascular smooth muscle cell migration by urotensin II. Naunyn Schmiedebergs Arch Pharmacol (2006) 373:381–6. 10.1007/s00210-006-0086-x 16896801

[149] Zhang Y-G Li J Li Y-G Wei R-H. Urotensin II induces phenotypic differentiation, migration, and collagen synthesis of adventitial fibroblasts from rat aorta. J Hypertens (2008) 26:1119–26. 10.1097/HJH.0b013e3282fa1412 18475149

[150] Zhang Y-G Kuang Z-J Mao Y-Y Wei R-H Bao S-L Wu L-B Osteopontin is involved in urotensin II-induced migration of rat aortic adventitial fibroblasts. Peptides (2011) 32:2452–8. 10.1016/j.peptides.2011.10.018 22036853

[151] Yi K Yu M Wu L Tan X. Effects of urotensin II on functional activity of late endothelial progenitor cells. Peptides (2012) 33:87–91. 10.1016/j.peptides.2011.11.016 22123628

[152] Federico A Zappavigna S Romano M Grieco P Luce A Marra M Urotensin-II receptor is over-expressed in colon cancer cell lines and in colon carcinoma in humans. Eur J Clin Invest (2014) 44:285–94. 10.1111/eci.12231 24372535

[153] Franco R Zappavigna S Gigantino V Luce A Cantile M Cerrone M Urotensin II receptor determines prognosis of bladder cancer regulating cell motility/invasion. J Exp Clin Cancer Res (2014) 33:48. 10.1186/1756-9966-33-48 24893613 PMC4061920

[154] Lecointre C Desrues L Joubert JE Perzo N Guichet P-O Le Joncour V Signaling switch of the urotensin II vasosactive peptide GPCR: prototypic chemotaxic mechanism in glioma. Oncogene (2015) 34:5080–94. 10.1038/onc.2014.433 25597409

[155] Coly P-M Perzo N Le Joncour V Lecointre C Schouft M-T Desrues L Chemotactic G protein-coupled receptors control cell migration by repressing autophagosome biogenesis. Autophagy (2016) 12(12):2344–62. 10.1080/15548627.2016.1235125 27715446 PMC5173274

[156] Bousette N Patel L Douglas SA Ohlstein EH Giaid A. Increased expression of urotensin II and its cognate receptor GPR14 in atherosclerotic lesions of the human aorta. Atherosclerosis (2004) 176:117–23. 10.1016/j.atherosclerosis.2004.03.023 15306183

[157] Vergura R Camarda V Rizzi A Spagnol M Guerrini R Calo’ G Urotensin II stimulates plasma extravasation in mice via UT receptor activation. Naunyn Schmiedebergs Arch Pharmacol (2004) 370:347–52. 10.1007/s00210-004-0991-9 15526105

[158] Gendron G Simard B Gobeil F Sirois P D’Orléans-Juste P Regoli D. Human urotensin-II enhances plasma extravasation in specific vascular districts in Wistar rats. Can J Physiol Pharmacol (2004) 82:16–21. 10.1139/y03-122 15052301

[159] Johns DG Ao Z Naselsky D Herold CL Maniscalco K Sarov-Blat L Urotensin-II-mediated cardiomyocyte hypertrophy: effect of receptor antagonism and role of inflammatory mediators. Naunyn Schmiedebergs Arch Pharmacol (2004) 370:238–50. 10.1007/s00210-004-0980-z 15549273

[160] Zhou C-H Wan Y-Y Chu X-H Song Z Xing S-H Wu Y-Q Urotensin II contributes to the formation of lung adenocarcinoma inflammatory microenvironment through the NF-κB pathway in tumor-bearing nude mice. Oncol Lett (2012) 4:1259–63. 10.3892/ol.2012.932 23226801 PMC3506729

[161] Cirillo P De Rosa S Pacileo M Gargiulo A Angri V Fiorentino I Human urotensin II induces tissue factor and cellular adhesion molecules expression in human coronary endothelial cells: an emerging role for urotensin II in cardiovascular disease. J Thromb Haemost (2008) 6:726–36. 10.1111/j.1538-7836.2008.02923.x 18284603

[162] Liang D Liu L Ye C Zhao L Yu F Gao D Inhibition of UII/UTR system relieves acute inflammation of liver through preventing activation of NF-κB pathway in ALF mice. PLoS One (2013) 8:e64895 10.1371/journal.pone.0064895 23755157 PMC3670940

[163] Artemenko Y Lampert TJ Devreotes PN. Moving towards a paradigm: common mechanisms of chemotactic signaling in *Dictyostelium* and mammalian leukocytes. Cell Mol Life Sci (2014) 71:3711–47. 10.1007/s00018-014-1638-8 24846395 PMC4162842

[164] Pierce KL Tohgo A Ahn S Field ME Luttrell LM Lefkowitz RJ. Epidermal growth factor (EGF) receptor-dependent ERK activation by G protein-coupled receptors: a co-culture system for identifying intermediates upstream and downstream of heparin-binding EGF shedding. J Biol Chem (2001) 276:23155–60. 10.1074/jbc.M101303200 11290747

[165] Roussos ET Condeelis JS Patsialou A Chemotaxis in cancer. Nat Rev Cancer (2011) 11:573–87. 10.1038/nrc3078 21779009 PMC4030706

[166] Richardson BE Lehmann R. Mechanisms guiding primordial germ cell migration: strategies from different organisms. Nat Rev Mol Cell Biol (2010) 11:37–49. 10.1038/nrm2815 20027186 PMC4521894

[167] Theveneau E Mayor R. Neural crest migration: interplay between chemorepellents, chemoattractants, contact inhibition, epithelial-mesenchymal transition, and collective cell migration. Wiley Interdiscip Rev Dev Biol (2012) 1:435–45. 10.1002/wdev.28 23801492

[168] Sadik CD Luster AD. Lipid-cytokine-chemokine cascades orchestrate leukocyte recruitment in inflammation. J Leukoc Biol (2012) 91:207–15. 10.1189/jlb.0811402 22058421 PMC3290425

[169] Bravo-Cordero JJ Hodgson L Condeelis J. Directed cell invasion and migration during metastasis. Curr Opin Cell Biol (2012) 24:277–83. 10.1016/j.ceb.2011.12.004 22209238 PMC3320684

[170] Rostène W Kitabgi P Parsadaniantz SM. Chemokines: a new class of neuromodulator? Nat Rev Neurosci (2007) 8:895–903. 10.1038/nrn2255 17948033

[171] Borsig L Wolf MJ Roblek M Lorentzen A Heikenwalder M Inflammatory chemokines and metastasis – tracing the accessory. Oncogene (2014) 33:3217–24. 10.1038/onc.2013.272 23851506

[172] Hembruff SL Cheng N. Chemokine signaling in cancer: implications on the tumor microenvironment and therapeutic targeting. Cancer Ther (2009) 7:254–67. 20651940 PMC2907742

[173] Lim SY Yuzhalin AE Gordon-Weeks AN Muschel RJ Targeting the CCL2-CCR2 signaling axis in cancer metastasis. Oncotarget (2016) 7:28697–710. 10.18632/oncotarget.7376 26885690 PMC5053756

[174] Maolake A Izumi K Shigehara K Natsagdorj A Iwamoto H Kadomoto S Tumor-associated macrophages promote prostate cancer migration through activation of the CCL22-CCR4 axis. Oncotarget (2017) 8:9739–51. 10.18632/oncotarget.14185 28039457 PMC5354767

[175] Kato T Fujita Y Nakane K Mizutani K Terazawa R Ehara H CCR1/CCL5 interaction promotes invasion of taxane-resistant PC3 prostate cancer cells by increasing secretion of MMPs 2/9 and by activating ERK and Rac signaling. Cytokine (2013) 64:251–7. 10.1016/j.cyto.2013.06.313 23876400

[176] Long H Xiang T Qi W Huang J Chen J He L CD133+ ovarian cancer stem-like cells promote non-stem cancer cell metastasis via CCL5 induced epithelial-mesenchymal transition. Oncotarget (2015) 6:5846–59. 10.18632/oncotarget.3462 25788271 PMC4467406

[177] Albertin G Guidolin D Sorato E Spinazzi R Mascarin A Oselladore B Pro-angiogenic activity of urotensin-II on different human vascular endothelial cell populations. Regul Pept (2009) 157:64–71. 10.1016/j.regpep.2009.04.006 19362580

[178] Morimoto R Satoh F Murakami O Totsune K Arai Y Suzuki T Immunolocalization of urotensin II and its receptor in human adrenal tumors and attached non-neoplastic adrenal tissues. Peptides (2008) 29:873–80. 10.1016/j.peptides.2007.06.025 17686550

[179] Takahashi K Totsune K Murakami O Arihara Z Noshiro T Hayashi Y Expression of urotensin II and its receptor in adrenal tumors and stimulation of proliferation of cultured tumor cells by urotensin II. Peptides (2003) 24:301–6. 10.1016/S0196-9781(03)00039-1 12668216

[180] Desrues L Lefebvre T Lecointre C Schouft M-T Leprince J Compère V Down-regulation of GABA(A) receptor via promiscuity with the vasoactive peptide urotensin II receptor. Potential involvement in astrocyte plasticity. PLoS One (2012) 7:e36319. 10.1371/journal.pone.0036319 22563490 PMC3341351

[181] Takahashi K Totsune K Murakami O Shibahara S. Expression of urotensin II and urotensin II receptor mRNAs in various human tumor cell lines and secretion of urotensin II-like immunoreactivity by SW-13 adrenocortical carcinoma cells. Peptides (2001) 22:1175–9. 10.1016/S0196-9781(01)00441-7 11445248

[182] Birker-Robaczewska M Boukhadra C Studer R Mueller C Binkert C Nayler O. The expression of urotensin II receptor (U2R) is up-regulated by interferon-gamma. J Recept Signal Transduct Res (2003) 23:289–305. 10.1081/RRS-120026972 14753294

[183] Wu Y-Q Song Z Zhou C-H Xing S-H Pei D-S Zheng J-N Expression of urotensin II and its receptor in human lung adenocarcinoma A549 cells and the effect of urotensin II on lung adenocarcinoma growth in vitro and in vivo. Oncol Rep (2010) 24:1179–84. 20878108 10.3892/or_00000970

[184] Balakan O Kalender ME Suner A Cengiz B Oztuzcu S Bayraktar R The relationship between urotensin II and its receptor and the clinicopathological parameters of breast cancer. Med Sci Monit (2014) 20:1419–25. 10.12659/MSM.890459 25112588 PMC4138065

[185] Cook J Hagemann T. Tumour-associated macrophages and cancer. Curr Opin Pharmacol (2013) 13:595–601. 10.1016/j.coph.2013.05.017 23773801

[186] Ryan CM Brown JAL Bourke E Prendergast ÁM Kavanagh C Liu Z ROCK activity and the Gβγ complex mediate chemotactic migration of mouse bone marrow-derived stromal cells. Stem Cell Res Ther (2015) 6:136. 10.1186/s13287-015-0125-y 26204937 PMC4603944

